# New data on settlement and environment at the Pleistocene/Holocene boundary in Sudano-Sahelian West Africa: Interdisciplinary investigation at Fatandi V, Eastern Senegal

**DOI:** 10.1371/journal.pone.0243129

**Published:** 2020-12-09

**Authors:** Benoît Chevrier, Laurent Lespez, Brice Lebrun, Aline Garnier, Chantal Tribolo, Michel Rasse, Guillaume Guérin, Norbert Mercier, Abdoulaye Camara, Matar Ndiaye, Eric Huysecom

**Affiliations:** 1 Archéologie et Peuplement de l’Afrique, University of Geneva, Geneva, Switzerland; 2 Department of Geography, University Paris Est Créteil, LGP-CNRS UMR 8591, Créteil, France; 3 Centre Européen de Recherche et d'Enseignement des Géosciences de l'Environnement, Aix-en-Provence, France; 4 Centre de recherche en physique appliquée à l’archéologie, UMR 5060 CNRS Institut de recherche sur les Archéomatériaux, Pessac, France; 5 UMR 5133 CNRS Archéorient, University Lumière Lyon 2, Lyon, France; 6 Archaeology laboratory, University Cheikh Anta Diop, Dakar, Senegal; Max Planck Institute for the Science of Human History, GERMANY

## Abstract

The end of the Palaeolithic represents one of the least-known periods in the history of western Africa, both in terms of its chronology and the identification of cultural assemblages entities based on the typo-technical analyses of its industries. In this context, the site of Fatandi V offers new data to discuss the cultural pattern during the Late Stone Age in western Africa. Stratigraphic, taphonomical and sedimentological analyses show the succession of three sedimentary units. Several concentrations with rich lithic material were recognized. An *in situ* occupation, composed of bladelets, segments, and bladelet and flake cores, is confirmed while others concentrations of lithic materials have been more or less disturbed by erosion and pedogenic post-depositional processes. The sequence is well-dated from 12 convergent OSL dates. Thanks to the dating of the stratigraphic units and an OSL date from the layer (11,300–9,200 BCE [13.3–11.2 ka at 68%, 14.3–10.3 ka at 95%]), the artefacts are dated to the end of Pleistocene or Early Holocene. Palaeoenvironmental data suggest that the settlement took place within a mosaic environment and more precisely at the transition between the open landscape of savanna on the glacis and the plateau, and the increasingly densely-wooded alluvial corridor. These humid areas must have been particularly attractive during the dry season by virtue of their rich resources (raw materials, water, trees, and bushes). The Fatandi V site constitutes the first stratified site of the Pleistocene/Holocene boundary in Senegal with both precise geochronological and palaeoenvironmental data. It complements perfectly the data already obtained in Mali and in the rest of western Africa, and thus constitutes a reference point for this period. In any case, the assemblage of Fatandi V, with its bladelets and segments and in the absence of ceramics and grinding material, fits with a cultural group using exclusively geometric armatures which strongly differs from another group characterized by the production of bifacial armatures, accompanied in its initial phase by ceramics (or stoneware) and grinding material.

## Introduction / Background

The end of the Palaeolithic represents one of the least known periods in the history of western Africa, both in terms of its chronology and the identification of cultural entities based on typo-technical analyses of its industries [1]. This is mainly due to the scarcity of stratified sites of the Pleistocene/Holocene transition and the Early Holocene. Very few sedimentary sequences have indeed been identified, as the great majority of them were probably eroded during the climatic optimum of the Holocene, and archaeological deposits presumed to belong typologically to the end of the Palaeolithic are rare. In cases where a site is assume to date from one of these periods, the archaeological material is often mixed with more recent remains, making it impossible to obtain an absolute date from either C14 or OSL [2].

**Fig 1:**
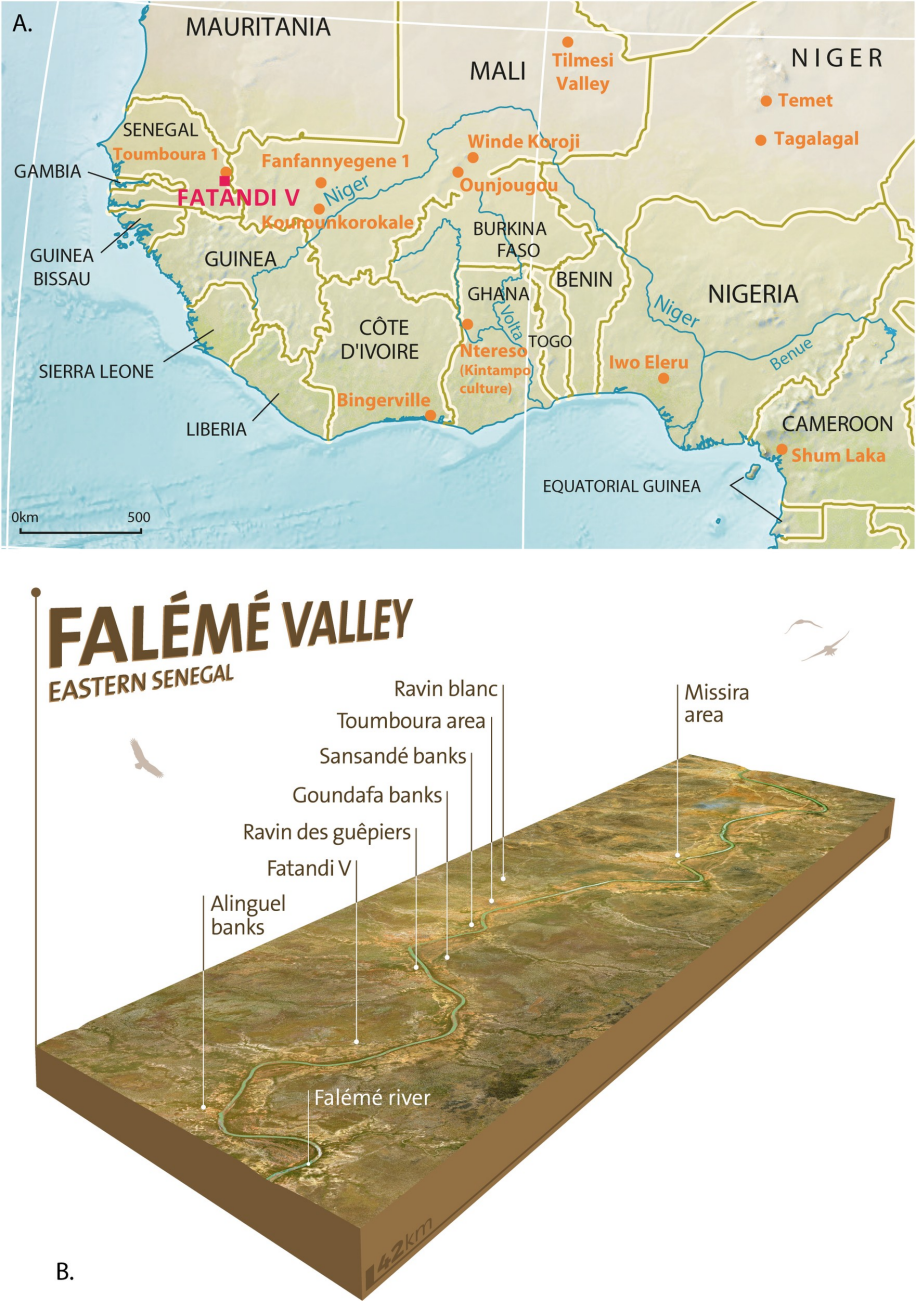
Western Africa archeological map. (A) Location of Fatandi V with surveyed or excavated archaeological occupations and sites mentioned in the text. (B) Location of Fatandi V and surveyed sites in Falémé valley (map design by Laboratory Archaeology and Population in Africa (APA) of the Department of Genetics and Evolution (University of Geneva) based on views from USGS Earth Resources Observatory and Science Center and CIA World Factbook).

Those rare stratified sites, both studied and dated, suggest highly contrasted regional evolutionary trajectories: the transition from nomadic Palaeolithic populations, with a foraging subsistence mode, to semi-sedentary proto-Neolithic societies practising intensive selective foraging, and then to sedentary Neolithic populations producing their own food, seems to be increasingly complex and variable depending on the region [3]. To give just one example, the populations of Ravin de la Mouche at Ounjougou in Mali already mastered both ceramics and bifacial knapping around the 10th millennium BCE [10th millennium cal BC], most probably in the context of the foraging of grasses [4] (for greater clarity and to unify chronological notations, we will indicate in the text the dates unified in BCE (before common era), then in brackets [] the dates originally produced by chronologists (radiocarbon: cal BC, OSL: ka etc.)). On the other hand, the populations of Iwo Eleru in Nigeria or of Shum Laka in Cameroon knew neither pottery nor bifacial point knapping, and essentially produced geometric tools (Fig 1A) [5,6].

Finally, the question of a parallel evolution over the entire Holocene period of two populations that may be identified either by the use of bifacial pointed tools, or by geometric and possibly microlithic tools, is again indirectly raised [1,7,8]. The present study provides new data from a recently discovered site, Fatandi V, to illustrate the complexity of late Palaeolithic cultural assemblages in western Africa, their chronology and the environmental context in which they evolved to the Neolithic.

## Site location and research history

### Site location

**Fig 2:**
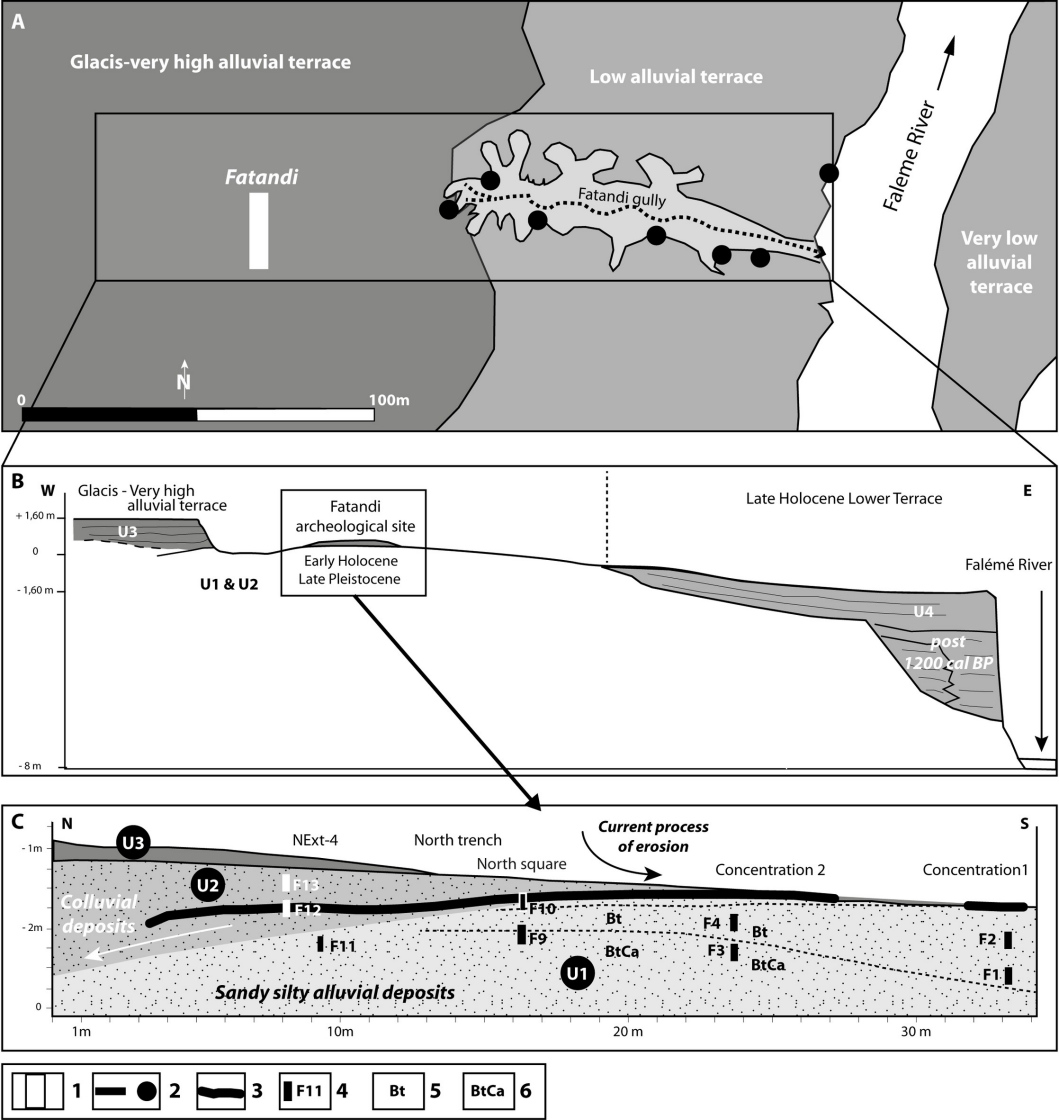
Geomorphological context of the archaeological site of Fatandi. (A) General situation; (B) Main stratigraphical and geomorphological units; (C) Detailed cross-section showing sedimentary units (U) and pedological subunits. Caption: 1. Excavated area; 2. Archeological and geomorphological sections; 3. Archaeological layer; 4. Location of OSL, phytolith and micromorphologic samples; 5. Illuvial horizon; 6. Carbonated illuvial horizon (figure designed by L. Lespez based on data from fieldwork).

The site is located on the left bank of the river Falémé, some 100 meters from the riverbank, between the villages of Alinguel and Goundafa (13°50’50.38”N / 12°09’55.92”W) [9] (Fig 1B). It is situated on a glacis terrace lying more than 8 meters above a low alluvial terrace and the river itself (Fig 2A and 2B). The sediment observed on this low terrace is composed of an alluvial facies (more or less organic fluvial silts: overbank deposits), sealed by colluvial sediments. The terrace may be attributed to the last millennia according to the available data and comparison with other observations made along the Falémé [10–13]. The current vegetation may be described as a wooded savanna with *Acacia seyal*, *Balanites aegyptiaca* and *Combretum sp*. as the dominant species.

**Fig 3:**
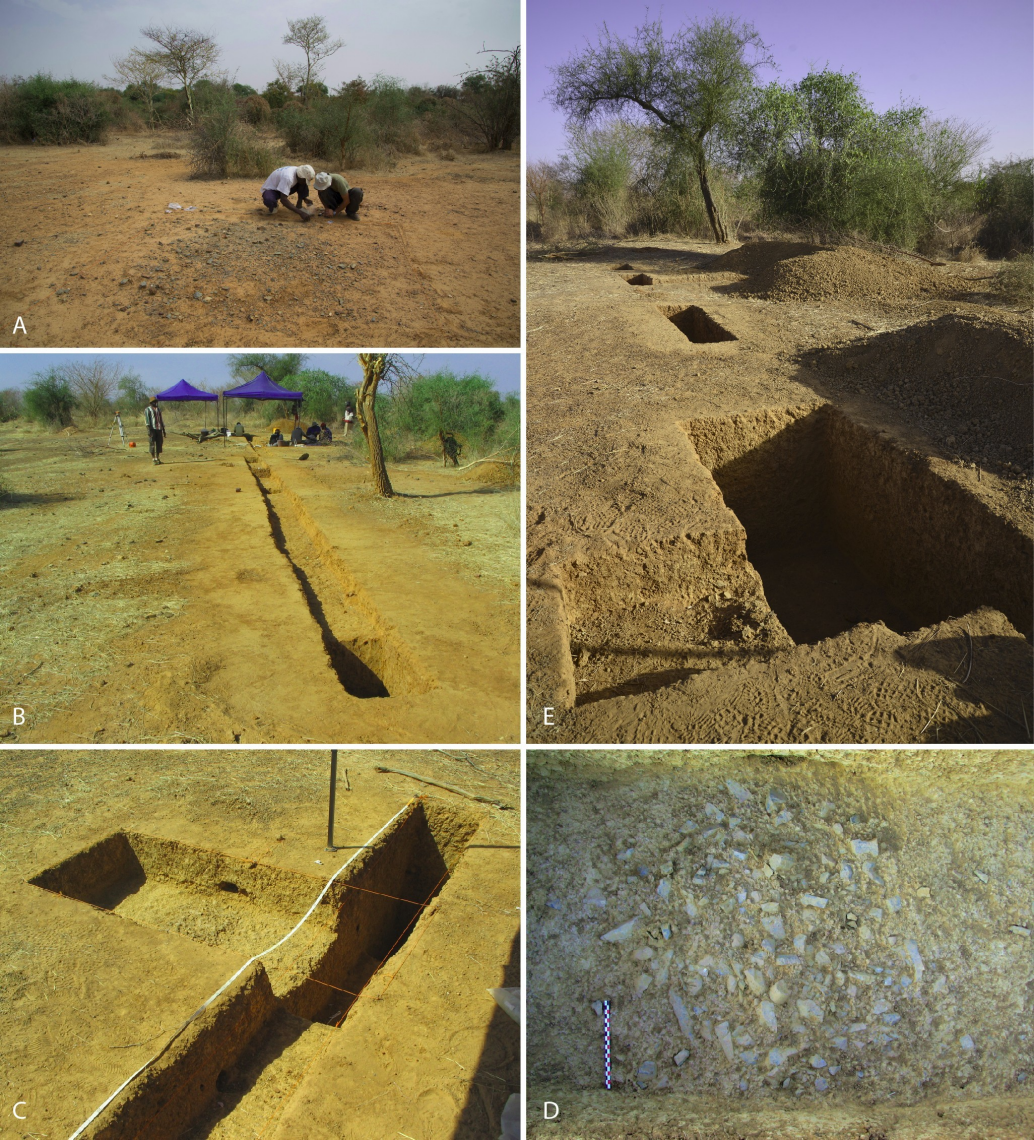
Photos of Fatandi V site and excavations. (A) Concentration 1, collecting artefacts in 2013. (B) North-south trenches, view from the south, 2013 field campaign. (C) North square during 2013 excavation. (D) Detail of the lithic concentration in North square during 2013 excavation. (E) North square in the foreground and North trench extension in the background at the end of 2014 excavation. All photos by B. Chevrier.

**Fig 4:**
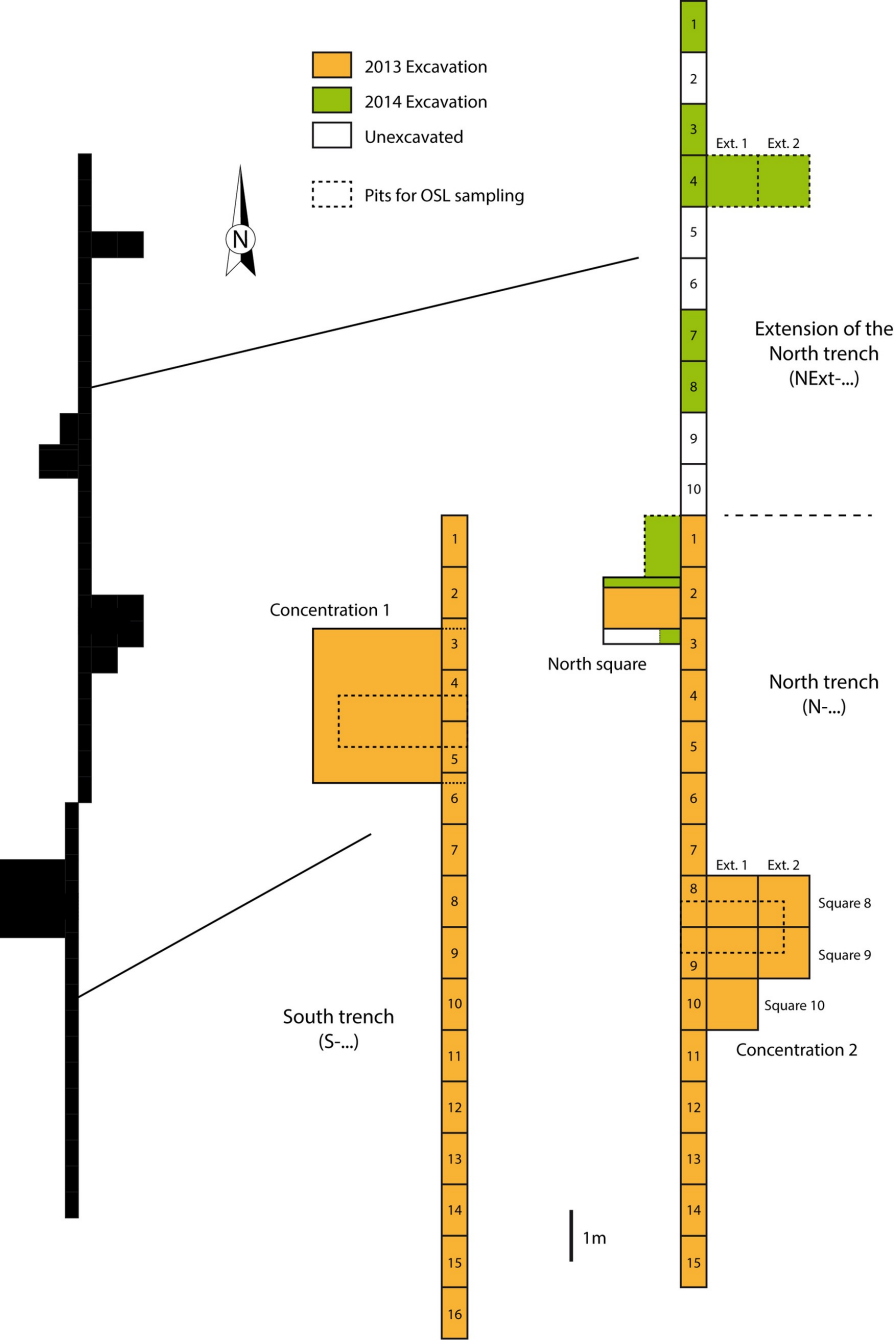
Location of the surface concentrations and excavated sectors during 2012 to 2014 field missions.

Discovered during surveys in 2012, the site consists of several more or less rich concentrations of lithic artefacts, deposited on a gentle SSW-facing slope (Fig 3A). Several ceramic sherds were spotted at the highest points of the glacis, corresponding to the summit and western zones. In the rest of the site, only lithics were collected and no organic remains were found. Multiple sectors were identified. An expansive and particularly rich concentration of material at the surface was named Concentration 1 (Figs 3A and 4). This sector lies to the south and is lower down on the slope. Another sector 12 meters to the north, known as Concentration 2 (Fig 4), presented more limited concentrations of lithic material currently undergoing erosion.

### Research history

Three field seasons were undertaken at Fatandi V, from 2012 to 2014. In 2012, attention was focused on the Concentration 1 sector to obtain preliminary techno-cultural information. This area is represented by a concentration of thousands lithic artefacts lying on surface on approximately 9 m^2^ (Fig 4). In 2013, lithics were methodically collected and their distribution precisely documented to discuss the site formation process (Fig 3A).

Subsequently, two north-south running trenches, connected but slightly offset from each other, were dug over a total surface of 31x 0.5 m^2^ (Figs 3B and 4). The South trench cut through the side of Concentration 1 in order to verify the apparent absence of objects in stratigraphy. The excavation of the South trench showed that there are only a few remaining objects in stratigraphy. Except these few items, the below sediments are sterile.

**Fig 5:**
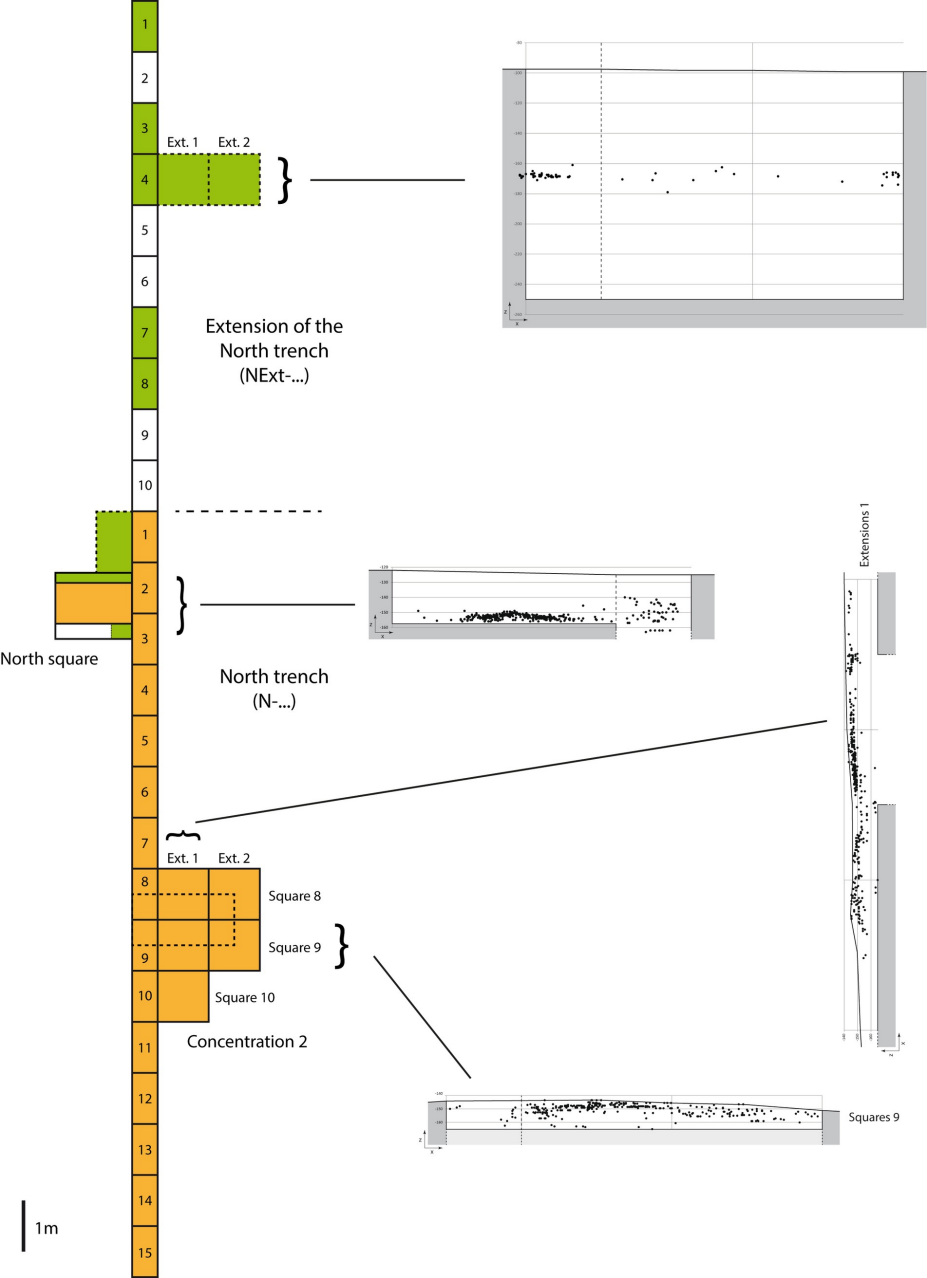
Artefact concentrations profiles in the North trench and its extension.

The North trench overlapped the sector of Concentration 2, with its artefacts partly on surface, and was extended towards the north in order to verify the stratigraphic position of the lithics (Figs 4 and 5). When it was observed that there were a quite large number of stratified artefacts, the Concentration 2 area was excavated in its totality over an area of 5 m^2^. This North trench also aimed at testing the archaeological and stratigraphic potential of the northern part, which is higher, as well as obtaining sediment samples for OSL dating. For the latter, two deeper excavations were dug to a depth of 1 m in both sectors Concentration 1 and Concentration 2. The two north-south trenches were dug to depths of between 10 and 40 cm, and several sections were dug some tens of centimeters deeper, to a maximum depth of 1.20 m, as a test.

Concentration 2 presents two sub-concentrations in the process of being eroded. The surface material was collected in its entirety and stratified artefacts longer than 2 cm were recorded. In addition, the smaller lithic fragments (< 2 cm long) were collected per ¼ m^2^. The excavation of the North trench sections as well as 5 m^2^ of the extension (Fig 4) confirmed the presence of artefacts in the archaeological level, well-contextualized within the stratigraphy. Below this level, no artefacts were recorded (Fig 5).

**Fig 6:**
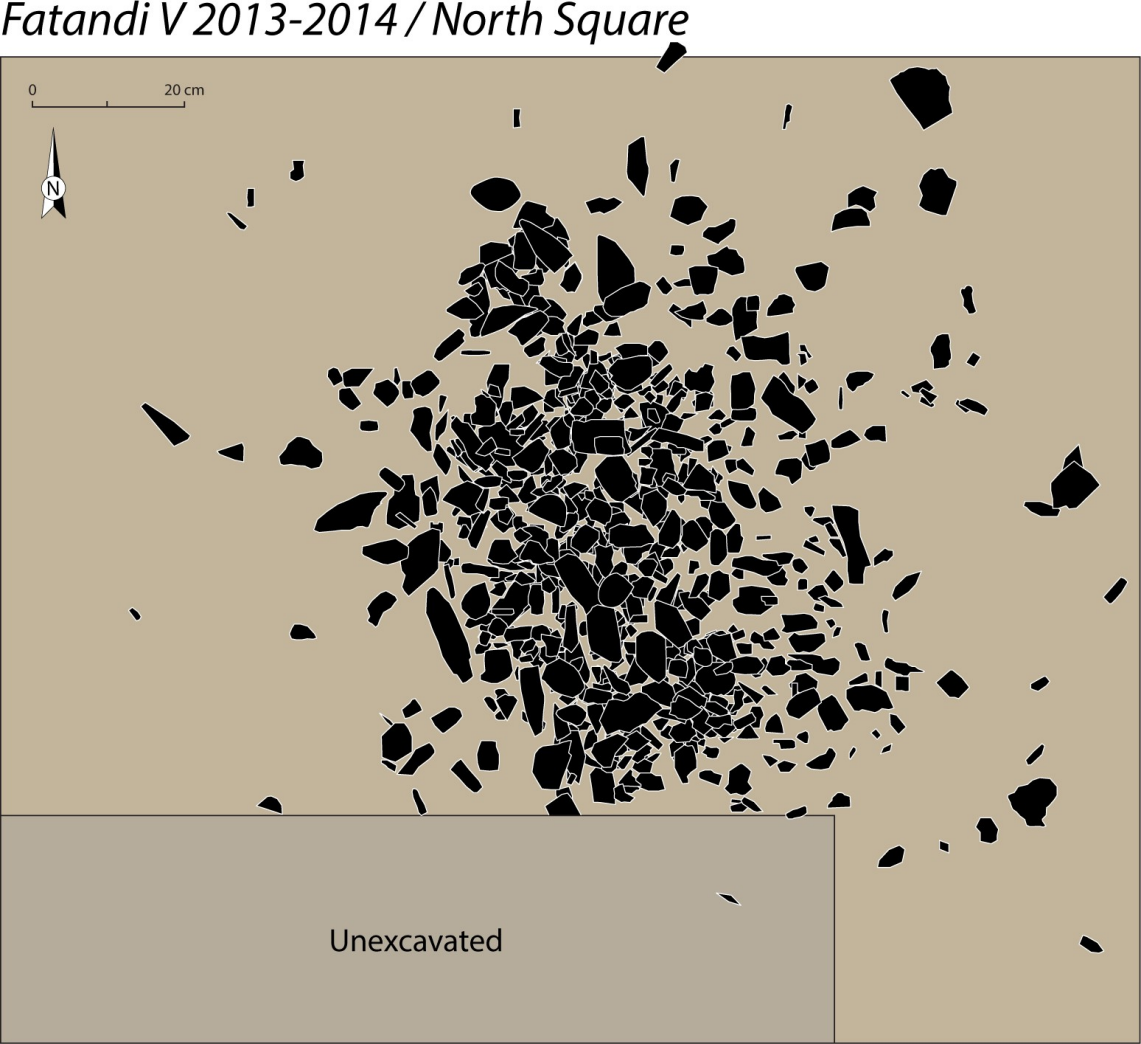
Drawing of the lithic concentration in the North square (drawing by B. Chevrier).

In addition, an extension called the North square was opened up off sections N-2 and N-3 (sections 2 and 3 of the North trench) (Figs 3C and 4) to obtain sediment samples for dating. Finally it delivered a significant concentration of *in situ* remains, measuring roughly 1m^2^ by 10 cm thickness (Figs 3D, 5 and 6). The North square concentration was finely excavated in 2013 and 2014.

In 2014, the operations undertaken in 2013 were completed and several additional extensions were investigated. These extensions reached in some areas a depth of 1.20 m in order to obtain OSL samples. The North trench was extended 10 m further north, with 5 additional sections of ½ m^2^ (Figs 3E and 4). An extension of 2 m^2^ was dug off section NExt-4 (section 4 of the North trench extension) (Fig 4) and excavated to a depth of some 1.50 m (Fig 5).

Only one archaeological level has been identified and well located in stratigraphy. It was being eroded in the Concentration 2 area and is still *in situ* in the northern part of the site (Fig 5). A multidisciplinary approach using geomorphological, palaeoenvironmental, taphonomical, geochronological and lithic analyses has been developed and has led to a global interpretation of the site and the archaeological deposits.

## Materials and methods

All necessary permits were obtained at the Fundamental Institute of Black Africa (IFAN) of the Cheikh Anta Diop University of Dakar (UCAD) for the described study which complied with all relevant regulations. The archaeological material is available for consultation after authorization from the Fundamental Institute of Black Africa (IFAN) of the Cheikh Anta Diop University of Dakar (UCAD) and the Laboratory Archaeology and Population in Africa (APA) of the Department of Genetics and Evolution of the University of Geneva (UniGE). The main part is stored at the Fundamental Institute of Black Africa (IFAN), Dakar, Senegal. A selection of pieces from North square, extracted for the study, is stored at the University of Geneva, Switzerland.

### Geomorphological method

Geomorphological and sedimentary investigations were developed following field observations, aided by descriptions of sections in the formations from the end of the Pleistocene and the Holocene. These allow us to propose a sedimentary architecture from the upper formation dominating the Falémé as well as a stratigraphic position for the archaeological remains. In parallel, the examination of the sediments strengthened the evidence for a succession of sedimentary layers and allowed us to take samples for laboratory analyses.

### Dating methods

In order to obtain chronological data for the site of Fatandi V, optically stimulated luminescence (OSL) dating was performed. This palaeodosimetric technique takes advantage of the grains of quartz present in sediments, which behave as a sort of stopwatch capable of recording the time elapsed since their last exposure to light [14]. This event generally corresponds to the deposition of the sedimentary unit in question.

At the site of Fatandi V, 12 sediment samples were taken in 2013 and 2014. During night time, samples were taken by scraping the stratigraphic section, taking care to eliminate from the offset the first centimeters of sediment exposed to the daylight.

The samples were taken from 5 distinct zones (Fig 2). Samples F1 and F2 were situated beneath Concentration 1, at 70 and 30 cm respectively from the current surface of the archaeological layer. Samples F3 and F4 were taken beneath Concentration 2, at 76 and 36 cm respectively from the current surface of the archaeological deposits. F9 and F10 come from the North square and were situated at 29 and 10 cm beneath the archaeological level. Samples F11, F12 and F13 were sampled in section 4 of the North trench extension, respectively 30 cm directly beneath, within and 30 cm above the archaeological layer. Finally, samples F5, F6, and F7 were sampled in the upper part of the site respectively at 85, 60, and 40 cm beneath the current surface.

The OSL dating method requires the determination of two quantitative variables: the annual dose or dose rate (Da, in Gy/ka) which corresponds to the energy absorbed by the quartz grains per unit of time, and the equivalent dose (De, in Gy) which corresponds to the total dose absorbed by the quartz grains during their buried life. Age is calculated as the ratio De/Da.

The ß dose rates were calculated according to the concentration of radioelements in the sediment (U, Th, and K series), using the conversion factors of concentration/dose outlined in Guérin *et al*. (2011) [15] and the attenuation factors linked to the size of the grains as outlined in Guérin *et al*. (2012) [16]. The concentrations of radioelements were determined by high resolution gamma spectrometry (broad germanium detector, Canberra). A correction of the calculated dose rate was also made to account for the water present in the sediment that absorbs part of the radiation, which is, as a result, no longer available for the irradiation of the quartz grains. To this end, a theoretical water concentration at saturation (W) was calculated from the granulometric distribution characteristic of each sample, following Nelson and Rittenour (2015) [17]. For the concentration of water in the sediment (expressed as a fraction F of the level of saturation, or WF), which furthermore varies over the course of time, an average value F of 50 ± 15% was estimated so as to sufficiently cover the range of water concentrations that could potentially have been prevailed since the sediment’s deposition.

The γ dose rate induced by the radiation was determined in the field with a portable γ-spectrometer Canberra Inspector 1000 (LaBr probe). A hole, 7 cm in diameter and 30 cm in depth, was bored into the section at the location of each sample in order to insert the probe. Each measurement allowed to record a spectrum of energy covering the range of natural gamma waves [18]. The processing of spectra was performed by way of energy threshold technique [19] and by using the calibration procedure of Miallier *et al*. (2009) [20]. The results were subsequently corrected with respect to the water concentration at the time of data acquisition.

The contribution of cosmic radiation to the total dose rate was calculated using the equation established by Prescott and Hutton (1994) [21], of which the primary parameter is the burial depth of the sample.

The OSL measurements, necessary for determining the De, were performed using quartz grains (100–140 μm) extracted from the sediments following a series of mechanical and chemical procedures, according to a method presented in Lebrun *et al*. (2016) [22]. All preparatory steps and measurements were performed under a red-orange light with no effect on the OSL signal. Two measurement techniques were employed: the multi-grain analyses were carried out with a Lexsyg Smart Reader [23] and the single-grain analyses with a Risø TL/OSL-DA-20 system [24]. In both cases, the grains were stimulated with a green light with a wavelength in the range of 525 nm.

**Fig 7:**
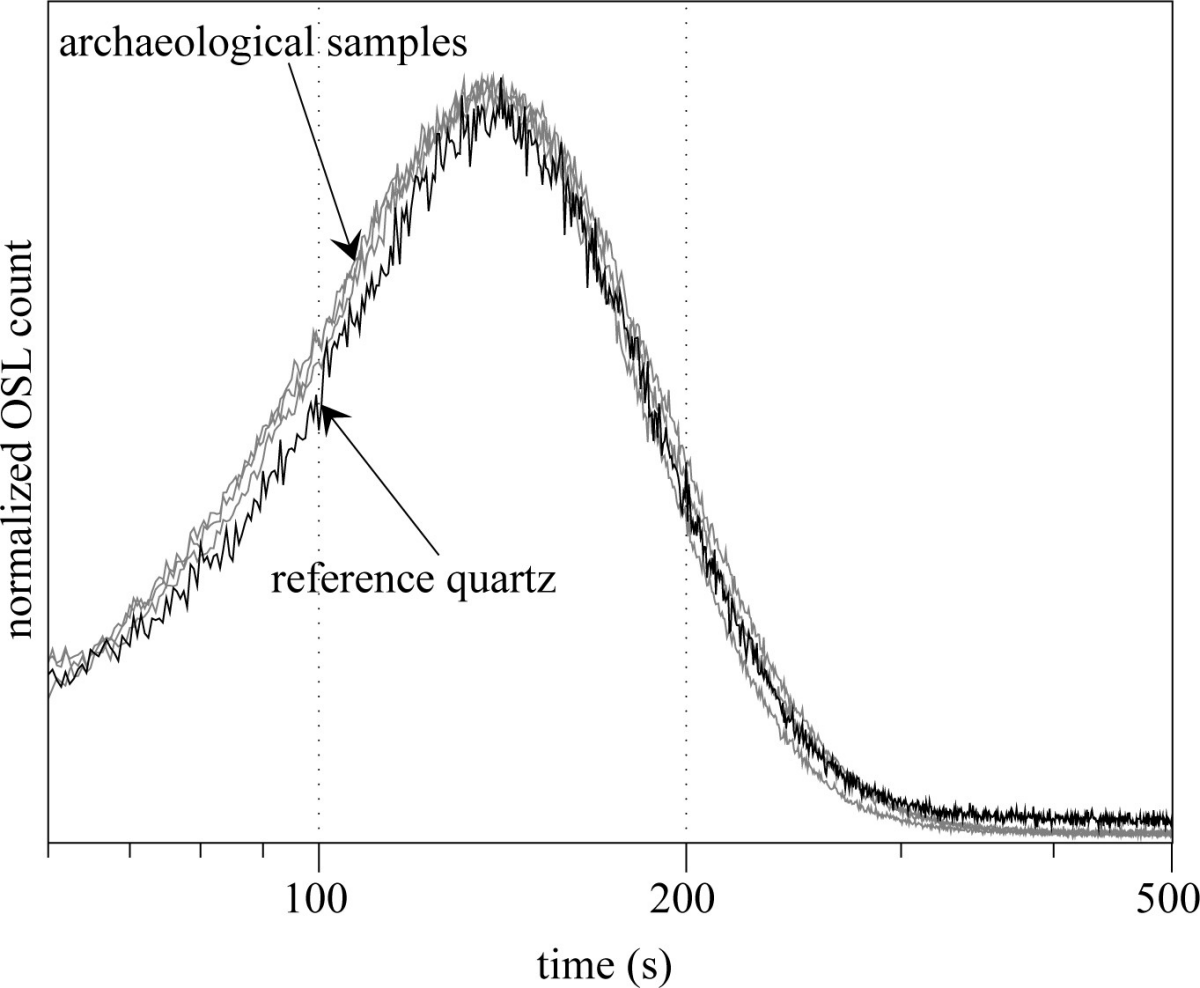
Comparison of the natural luminescence signals in LM-OSL mode for samples F11, F12, and F13 with a reference quartz [26] (signal generated by irradiation ß of 20 Gy). The signal is measured over a period of 2,000 s (here only the first 500 seconds are shown), while the strength of the electroluminescence diodes is risen linearly from 0 to 60 mW/cm^2^. It is worth noting that the signal is dominated by the fast component and is thus adapted to OSL dating by the SAR protocol.

A depletion ratio test [25], performed on all samples, provided confirmation for the global absence of feldspars. A study of the luminescence signals was also undertaken in order to ensure that the samples were dominated by the fast component. In order to do this, several multi-grain discs were analyzed by linearly modulating the strength of the OSL electroluminescence stimulation diodes (from 0 to 60 mW/cm^2^ over 2,000 s). The readings obtained (LM-OSL) were subsequently compared to those of quartz provided by the Nordic Laboratory for Luminescence Dating (Risø) [26], dominated by the fast component. As we can see in Fig 7, the fast component also dominates the signals from the quartz of Fatandi V, which allows us to consider the single aliquot and regenerative dose protocol (SAR [27,28]) for measuring the De_s_ of these samples (Fig 7).

**Fig 8:**
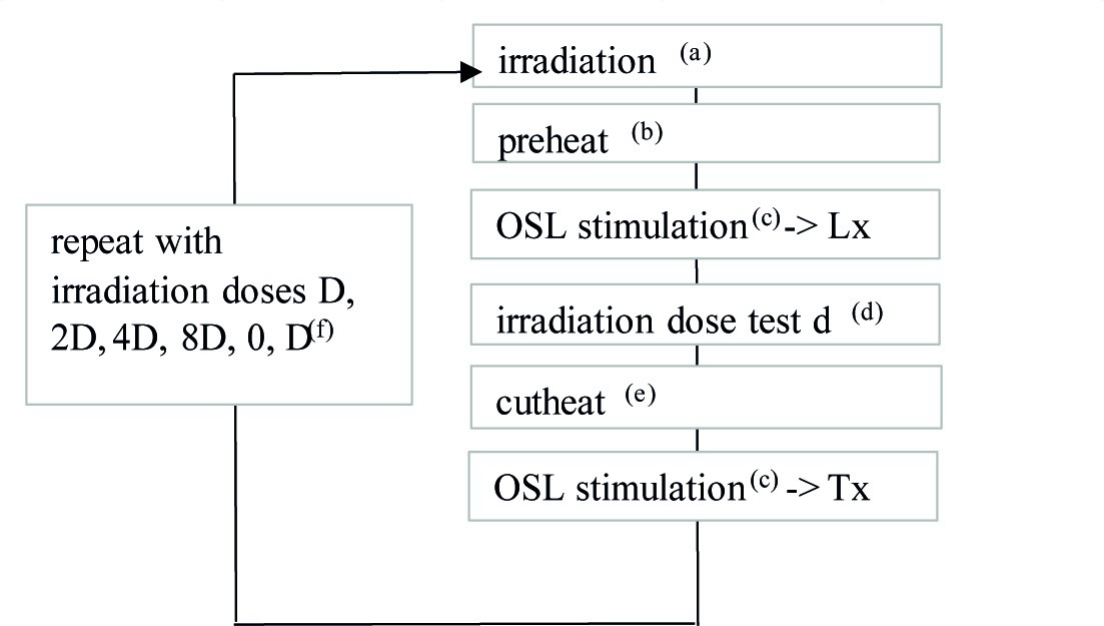
SAR protocol applied to the samples from Fatandi V. (a) For the first measurement cycle, no dose was applied. (b) The preheats tested range from 200 to 280°C, maintained for 10 s. (c) The stimulation lasts 100 s for the multi-grains, 1 s for the single-grains; the signal and the background noise selected in order to calculate Lx and Tx are calculated respectively for the first 0.3 and last 0.5 seconds in multi-grain mode, and the first 0.17 and last 0.15 seconds in single-grain mode. (d) The test dose was set at 5 Gy for all samples. (e) The preheat (cutheat) after application of the test dose is 160°C, not maintained. (f) D is chosen in order so that the equivalent estimated dose for the first batch of aliquots is roughly equivalent to 3D.

Fig 8 shows the SAR protocol applied to the samples. The growth curve for the dose is modelled using the exponential saturation function Lx/Tx = a*(1-exp((D-b)/D_0_), where D is the dose, Lx/Tx is the normalized signal, and a, b, and D_0_ are the curve parameters (Fig 8).

The analysis of the data was performed with the Analyst program v4.57 [29]. For each aliquot, the selection criteria were applied based on the values of the recycling ratio (within ±10% of the unity) and recovery ratio (< 5% of the natural signal) as well as on the sensitivity of the signal (first test dose signal > 3 x background). The BayLum package was then used for the Bayesian analysis of the luminescence data (S1 File) [30–33].

The independence of the De with regards to the conditions of preheating were tested in the multi-grain mode. For each sample, the preheat temperature was varied between 200 and 280°C for a duration of 10 s. The preheat temperature for the test dose (*cutheat*) was fixed at 160°C for 0 second.

Some dose recovery tests (DRT) were also performed, in both multi-grain and single-grain mode, so as to test the capacity of the SAR protocol and the parameters chosen here to recover a dose administered in the laboratory (acceptability interval: [0.9;1.1]). The results of these tests and some of the measurements of equivalent doses are presented in OSL results section.

### Palaeoenvironmental reconstruction

For each of the OSL dates, the sediments were sampled in view of granulometric and phytolithic analyses. In parallel and within the same level, blocks of sediment were sampled so as to carry out micromorphological analyses. The blocks sampled were wrapped in plastered bandages in order to preserve the sediment structure. They were subsequently reinforced and reduced, and thin sections of large dimensions (12 by 6 cm) prepared following the traditional technique [34]. In total, 8 thin sections sampled from above (F13), below (F1, F2, F3, F4, F10) and at the archaeological layer (F12) were observed under a petrographic microscope in accordance with the methods proposed by Bullock *et al*. (1985) [35]. Micromorphological analyses were employed in order to understand the organization of micro-sedimentary layers and to detect pedological characteristics (humification, biological activity, micro-aggregation, hydromorphic features, oxydation, and desiccation features).

Phytoliths analysis was conducted on 8 samples collected in sediments beneath, within and above the archaeological layer. Due to their siliceous structure, phytoliths offer new perspectives for reconstructing ancient vegetation in semi-arid and fluvial environments [36–38]. Moreover, phytoliths are especially useful in open environments as they help to determine the composition of the grass cover layer. Numerous studies conducted on African modern analogues have demonstrated the possibility of distinguishing several grass sub-families and thus to extract information on palaeoecological conditions in fossil assemblages [39–43]. Methods of phytolith extraction and analysis are those in Neumann *et al*. (2009) [37] and Garnier *et al*. (2013) [38]. Diagnostic phytolith morphotypes were described using the ICPN classification [44] and classified into three main categories: sclereids and globular morphotypes, attributed to woody and herbaceous dicotyledons; GSCP (Grass Short Cells Phytoliths), produced by Poaceae while some specific morphotypes correspond to non-grass monocotyledons (Arecaceae, Commelinaceae and Cypercaeae). Absolute phytolith counts range from 241 to 318 diagnostic morphotypes.

### Site formation reconstruction (taphonomy)

The different states of conservation of the lithic concentrations have led to the collection of more or less detailed taphonomic information depending on the sector in order to understand the formation, erosion and movement of the archaeological level.

In 2012 the discovery of the Concentration 1 composed of lithic objects on a surface of about 9 m^2^ led us to make photographic records of this group of remains. We also undertook a succinct study of their distribution, followed by a preliminary analysis of 20 pieces found at the surface, including cores, negative blanks [45] and flakes of interest.

In 2013, this area of 9 m^2^ of Concentration 1 was photographed systematically and in detail in order to produce high-definition images (7,000 px wide), allowing us to preserve an accurate record of the organisation of the concentration prior to sampling. A grid was put in place to record the archaeological material per ^1^/_16_ m^2^ and produced information for 144 squares of 25 by 25 cm each (Fig 3A). All artefacts were collected within these squares, as well as from the vicinity, and were indiscriminately counted. Moreover, those that were shorter than 2 cm were also weighed to obtain a weight/number ratio and refine the distribution of small lithics that may be suspected of wider movements on surface. This process was repeated over the concentration in units of ^1^/_16_ m^2^. The 20 objects documented in 2012 were relocated in order to analyze their displacement between 2012 and 2013. This was made possible through the re-identification of one of these artefacts, considering the nails used as markers had disappeared. This artefact was chosen as a reference mark for its proximity to the center of the concentration. The location of the 19 other pieces was recorded with reference to this artefact.

The excavations conducted in 2013 and 2014 made it possible to accurately record the stratigraphic position of the artefacts in the areas where the pieces are still buried, or to observe the absence of lithics in certain areas. We have also kept information on the orientation and dip of the artefacts. This data would allow for fabric analysis, only on stratified artefacts in Concentration 2 and northern areas of the site, and refine the interpretation of burial process in Fatandi V [46,47]. However, a large number of Concentration 2 lithics were already eroded and the northern excavation area, in particular North square, is small (Figs 4 and 5). The value of a detailed fabric analysis for understanding site formation would therefore be currently limited. A larger excavated area with a completely stratified occupation would be required and possible in Fatandi V (the corner of a lithic concentration may have been discovered in the extension of the North trench). Moreover, as regards North square dense concentration, a microstratigraphic study is absolutely necessary to understand the abandonment and postdepositional processes. In the current state of excavation, the stratigraphic and geological data and the information about artefact displacement were primarily used to discuss the site formation and the features of the erosion process.

### Archeological analytical method

The archaeological study focuses on the material from the North square. However, it is also useful to specify the number of objects longer than 2 cm recorded in the other sectors (Table 1).

**Table 1:** Count of artefacts longer than 2 cm, in surface for Concentration 1, in stratigraphy for excavated areas. All artefacts smaller than 2 cm were collected by area, but were not systematically quantified for the current study.

Archaeological area	Artefacts longer than 2 cm
Concentration 1 (surface artefacts)	1,180
Concentration 2 (Tr.8-9-10 extensions / stratified artefacts)	471
North trench (sections N-1 to N-15 / stratified artefacts)	114
North trench–extension (sections Next-4, Next-4 extensions 1 and 2, Next-7 / stratified artefacts)	61
North square (+ Ext-N, Ext-S / stratified artefacts)	729

In all the excavated areas, lithics smaller than 2 cm long were collected per ¼ m^2^ and stored pending future taphonomical and archaeological studies.

The North square was chosen for the study because of its artefact density, its taphonomic integrity and its well-stratified location (Figs 3D, 5 and 6). The lithic concentration of the North square is quantitatively representative of the excavated areas. The sample studied in this article consist of 729 artefacts longer than 2 cm. The lithic analysis detailed here has been completed by preliminary observations made on artefacts from the other sectors, in particular in the North trench in which three pieces are cores, and on surface artefacts [48].

The analysis is based on a classic technological approach of the lithic artefacts, aiming to reconstruct the *chaînes opératoires* and to consider evidence for the various objectives of lithic production (management of the raw materials, potential preparation of the nodules, knapping, production of blanks, potential recycling, tool production and use). This approach focuses on the study of the knapping traces on blocks and blanks, their order and relationships, in order to understand the history, the origin and the role of each artefact [49–52].

Given the large number of natural pieces and pieces with uncertain knapping traces (323 objects, i.e. 44.3% of the corpus), the simplicity of the production methods and the difficulty of precisely linking the blanks to core types, we opted for the qualitative collection of data. Moreover, the low number of bladelets for example (38 artefacts) makes a quantitative approach relatively imprecise, which would be more informative on a larger corpus. A future study could integrate artefacts from Concentration 2 and those from a more extensive excavation and could provide metric attributes.

Artefact refittings could be identified but were not systematically searched for. This preliminary information on the links between artefacts provided additional qualitative data about the *chaînes opératoires*.

## Geomorphology and stratigraphy

The examination of the sections from the excavations, as well as from sections observed in the Fatandi gully, allows an accurate reading of the stratigraphy, which consists of a succession of four main stratigraphic units (Fig 2). The first (U1) forms the base of the archaeological site. This unit is represented by white to grey light sandy-clay silts. The second (U2) starts in the area of the archaeological site and is thicker towards the north. This unit is represented by whitish-grey/light ocre-colored silty-sand sediments, less clayey, and constitutes a sedimentary prism that seems to have formed in the opposite direction to the dominant slope of the current topography, indicating a slope to the north at the moment of deposition of the colluvial sediments. The third stratigraphic unit (U3) corresponds to ocre-colored silts and fine sands. These form the upper pedosediments of the ancient glacis currently being eroded and, while they reach a depth of close to 1 m in the west, they only cover the archaeological levels of Fatandi to a depth of 10 cm. These layers were eroded following the incision of the Falémé to a depth of several meters before the alluvial sediments of the lower terrace (U4) were deposited, which themselves were covered by colluvial sediments resulting principally from the erosion of the glacis sediments.

The stratigraphic analysis thus indicates a location of artefacts either on the eroded surface of U1 (Concentration 1), or in stratigraphy at the interface of U1/U2 (Concentration 2, one part of the North trench and the North square), or within the colluvial context of U2 (extension of the North trench). U1 itself remains archaeologically sterile (Figs 2 and 5).

## OSL results

### Location of samples in stratigraphic units

As a reminder, F1, F2, F3, F4, F9, F10 and F11 samples come from U1, F12 and F13 from U2, F5 to F7 from U3. F1 and F3 were sampled 70 and 76 below the current surface, where the archaeological layer was outcropping. F2, F4, F9, F11 and F10 were located between 36 and 10 cm below the archaeological horizon. F12 was taken within the archaeological horizon, while F13 was taken 30 cm above it (Fig 2).

### Determination of the annual dose (Da)

The study of the activity of several radioactive elements present at the “beginning” (^238^U determined according to the readings for ^234^Th, ^234m^Pa, and ^235^U), in the “middle” (^226^Ra determined from ^214^Pb and ^214^Bi) and at the “end” of the chain (^210^Pb) of the series of uranium (^238^U) did not reveal any significant radioactive disequilibrium. Therefore, we subsequently considered the β dose rates determined from current concentrations to be representative of the past β dose rates (S1 Fig). Note that the set of samples present together a strong homogeneity in their concentrations of radioactive elements. On average, they contain around 1% potassium, 3 ppm uranium and 10 ppm thorium (Table 2).

**Table 2:** Data relevant for the calculation of the dose rate.

sample	burial depth (m)	Radionuclid content						Particle size distribution (%)			Water content (%)			Annual dose rate (Gy/ka)							
		U (ppm)		Th (ppm)		K (%)		Clay	Silt	Sand	W.F.		W	β		γ		cosmic		total	
**F1**	0.7	3.27	±0.03	9.69	±0.08	1.01	±0.01	21	62	17	0.11	±0.03	0.22	1.19	±0.01	0.88	±0.02	0.19	±0.02	2.26	±0.15
F2 *	0..3	3.39	±0.03	9.45	±0.09	0.94	±0.01	20	58	22	0.11	±0.03	0.22	1.15	±0.01	0.85	±0.02	0.19	±0.02	2.18	±0.15
**F3**	0..76	3.52	±0.03	9.54	±0.08	1.00	±0.01	14	64	21	0.11	±0.03	0.21	1.22	±0.01	0.88	±0.02	0.19	±0.02	2.29	±0.15
**F4**	0..36	2.87	±0.02	9.97	±0.08	1.05	±0.01	21	67	11	0.11	±0.03	0.22	1.18	±0.01	0.88	±0.02	0.22	±0.02	2.28	±0.15
F5 *	0..9	3.50	±0.03	10.45	±0.08	1.15	±0.01	20	71	9	0.12	±0.03	0.23	1.31	±0.01	0.93	±0.02	0.18	±0.02	2.41	±0.17
**F6**	0..6	3.05	±0.02	10.12	±0.08	1.10	±0.01	19	66	15	0.11	±0.03	0.22	1.24	±0.01	0.87	±0.02	0.19	±0.02	2.30	±0.13
**F7**	0..4	3.27	±0.03	9.71	±0.09	1.04	±0.01	19	65	16	0.11	±0.03	0.22	1.21	±0.01	0.82	±0.02	0.21	±0.02	2.24	±0.15
F9 *	0..3	2.93	±0.04	9.55	±0.12	1.02	±0.02	3	74	23	0.13	±0.04	0.25	1.14	±0.01	0.85	±0.02	0.18	±0.02	2.18	±0.15
**F10**	0..39	3.53	±0.05	9.46	±0.13	1.07	±0.03	7	76	17	0.13	±0.04	0.25	1.24	±0.02	0.88	±0.02	0.22	±0.02	2.34	±0.17
**F11**	0..98	2.78	±0.05	10.14	±0.14	1.07	±0.03	16	66	18	0.11	±0.03	0.23	1.18	±0.02	0.89	±0.02	0.17	±0.02	2.24	±0.16
**F12**	0..7	3.10	±0.05	9.53	±0.12	1.06	±0.03	12	69	19	0.12	±0.03	0.23	1.20	±0.02	0.89	±0.02	0.18	±0.02	2.27	±0.16
**F13**	0..38	3.25	±0.05	9.89	±0.14	1.07	±0.03	21	62	17	0.12	±0.03	0.23	1.23	±0.02	0.89	±0.02	0.21	±0.02	2.33	±0.16

W.F.: Estimated water concentration during burial; W: Water concentration at saturation (in % mass). The intervals for the granulometric categories considered here are: 0 to 3.9 μm (clay), 4 to 62.9 μm (silt), and 63 to 200 μm (sand). The dose rates presented here take into account water absorption. The β and γ dose rates are presented alongside their statistical errors. The cosmic dose rates and the total dose rate are given with their total errors (statistical and systematic). The samples with an asterix are those that were processed by Lebrun *et al*. (2016) [22] and mentioned here as a reminder.

Taking into account these parameters, the β dose rates adjusted for the absorption by water range from 1.14 ± 0.01 to 1.31 ± 0.01 Gy/ka (statistical errors; Table 2). For the calculation of the total dose rate, a systematic error of 10% for the U, Th, K concentrations (due to the calibration of the detector) was applied on top of the uncertainty mentioned above. The β dose rate represents between 48 and 63% of the total dose rates.

The γ dose rates obtained from the field probe and corrected for the water concentration at the moment of data acquisition range from 0.82 ± 0.02 to 0.93 ± 0.02 Gy/ka (statistical errors; Table 2). For the calculation of the total dose rates, a systematic error of 5% (due to the calibration of the detector) is taken into consideration.

For the calculation of the cosmic dose rates, we used the current burial depths. This is indicative because the burial depth has varied over the course of time through periods of accumulation and erosion. In addition, an uncertainty of 10% was added to the cosmic dose rates, which represent in all samples between 0.17 and 0.22 Gy/ka (Table 2). It is worth noting that these dose rates, although tainted by a high uncertainty, do not represent at the most more than 10% of the total dose rates.

The total dose rates therefore range between 2.18 ± 0.15 and 2.41 ± 0.17 Gy/ka and are relatively homogenous (RSD: 3%; Table 2).

### Determination of the equivalent dose (De)

The tests performed show that the temperature variation within the range considered (200–280°C) during the preheat does not significantly influence the value of De (S2 Fig). One temperature of 260°C was thus chosen for the remainder of the measurements. Table 3 presents the dose recovery ratio (DRR) obtained for each sample in both single and multi-grain mode. Multiple statistical models were tested: the simple arithmetic average (single-grain and multi-grain), which does not take into account individual errors; the “Central Age Model” based on a log-normal distribution (CAM) [53] (single-grain); and the Bayesian models, baSAR-Normal and baSAR-LogNormal_M, based respectively on a normal (gaussian) distribution and on a log-normal distribution (value of interest: median) (S1 File) [30,31,33]. The DRR remain within the interval [0.96;1.04] no matter which statistical model is employed for ten out of the twelve samples, suggesting that the measurement conditions and the statistical models are adequate, at least, for these ones in the context of tight distributions. The single-grain DRR from F6 and F7 are higher, close to 1.10. These latter are nonetheless judged to be acceptable.

**Table 3:** Results obtained for the dose recovery tests.

sample	multi-grain			single-grain											
	arithmetic mean			CAM						arithmetic mean		baSAR-Normal		baSAR-LogNormal_M	
	N	ratio		N	n	ratio		OD (%)		ratio		ratio		ratio	
F1	3	0.98	±0.02	300	57	1.02	±0.01	2	±2	1.00	±0.02	1.02	±0.01	1.02	±0.01
F2 *	5	1.02	±0.01	300	67	0.98	±0.01	4	±2	0.99	±0.01	0.97	±0.02	0.97	±0.02
F3	3	0.97	±0.02	300	69	0.99	±0.01	6	±1	0.99	±0.02	1.00	±0.01	0.99	±0.01
F4	3	0.96	±0.02	300	67	1.01	±0.01	3	±2	1.04	±0.02	1.02	±0.01	1.02	±0.01
F5 *	3	1.01	±0.02	300	55	1.00	±0.02	7	±2	1.02	±0.02	1.00	±0.02	1.00	±0.02
F6	3	0.97	±0.03	300	83	1.10	±0.01	7	±1	1.13	±0.02	1.11	±0.01	1.10	±0.01
F7	3	0.97	±0.01	300	57	1.08	±0.01	5	±1	1.09	±0.01	1.08	±0.01	1.08	±0.01
F9 *	-	-		300	56	0.99	±0.01	4	±2	1.00	±0.02	1.00	±0.01	1.00	±0.01
F10	3	0.98	±0.02	200	45	1.02	±0.01	0		1.07	±0.02	1.03	±0.02	1.02	±0.01
F11	3	0.97	±0.02	300	65	1.02	±0.01	5	±1	1.01	±0.02	1.02	±0.01	1.02	±0.01
F12	10	0.96	±0.02	500	66	1.01	±0.01	7	±1	1.00	±0.02	1.03	±0.01	1.03	±0.01
F13	3	0.99	±0.01	500	67	1.01	±0.01	0		1.01	±0.01	1.01	±0.01	1.01	±0.01

N: number of grains or aliquots measured; n: number of grains retained in the distribution, i.e. those that have passed the selection criteria (see main text); OD: overdispersion; CAM: Central Age (or dose) Model; baSAR-Normal and baSAR-LogNormal_M: Bayesian models based respectively on the normal (gaussian) distribution and log-normal taking into account the median. We give here the Bayes estimator as well as a calculated error of 68% from the credibility interval. *: data presented in part in Lebrun et al. 2016 [22].

**Fig 9:**
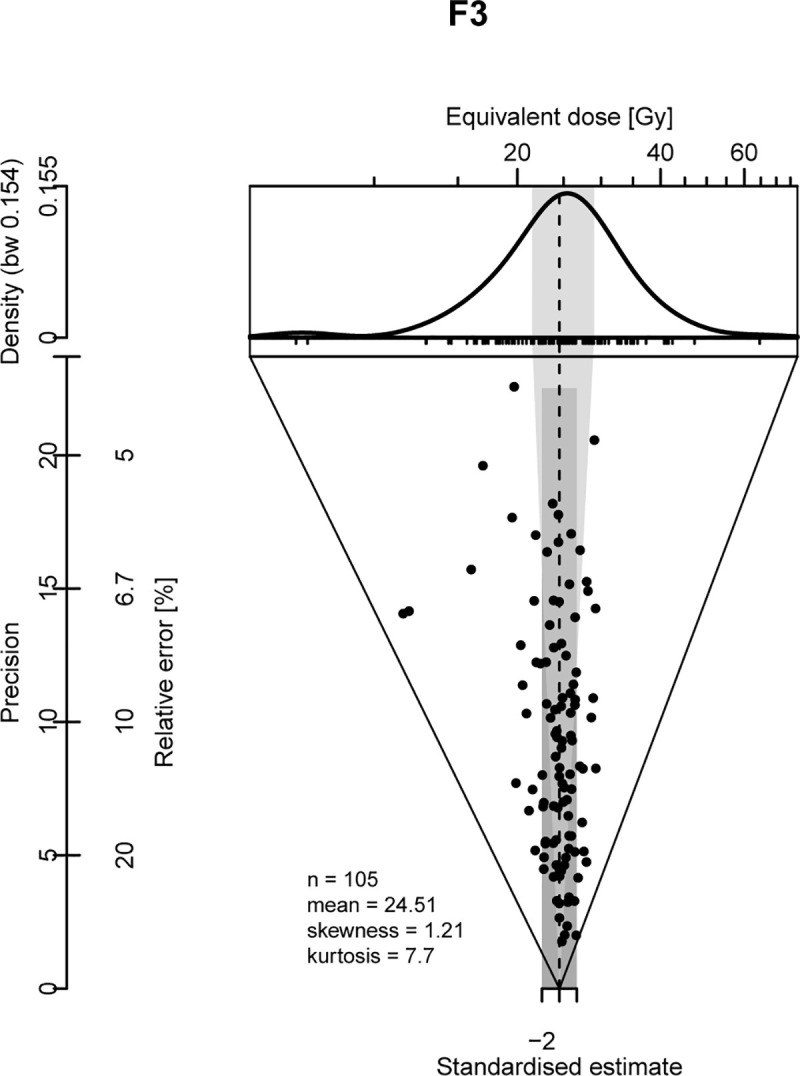
Abanico plot [55] for sample F3, in multi-grain mode.

Fig 9 presents an example of the single-grain equivalent dose distribution (F3). For this sample, as for the others from Fatandi V, no dependence on the central De (CAM) with the D_0_ saturation parameter was observed [54], the central De being relatively low, in the region of 10 to 20 Gy (Fig 9).

The De calculated with different statistical models are presented in Table 4. It is worth noting that there are no significant differences between the single and multi-grain De calculated with the CAM model. Some authors (e.g. [56]) have suggested that the discrepancy between the single and multi-grain De could be one of the criteria (though not the only one) allowing for the detection of a mixture of grain populations. The reverse might not be true: a statistical agreement of the De_s_ is not necessarily proof of absence of a mixture. In addition, other observations suggest that the distributions of De_s_ in the samples from Fatandi V could indeed result from a mix of populations. In other words, the CAM is perhaps not the most relevant model for our samples [57].

**Table 4:** Data relevant to the calculation of the equivalent dose for each sample according to different statistical models, without stratigraphic constraints.

	multi-grain					single-grain											
	CAM					CAM						arithmetic mean		baSAR-Normal		baSAR-logNormal_M	
sample	N	De (Gy)		OD (%)		N	n	De (Gy)		OD (%)		De (Gy)		De (Gy)		De (Gy)	
F1	30	24.3	±0.5	10	±1	400	99	25.2	±1.2	47	±4	28.2	±1.5	27.3	±1.1	25.6	±1.0
F2 *	51	22.8	±0.8	23	±2	500	145	22.5	±0.8	42	±3	23.9	±0.9	23.8	±0.8	21.5	±1.0
F3	30	24.4	±0.7	15	±2	400	107	23.3	±0.7	28	±2	24.5	±0.8	24.3	±0.7	23.6	±0.8
F4	31	24.7	±0.6	14	±2	400	109	24.0	±0.6	25	±2	26.0	±1.1	25.9	±0.7	25.4	±0.8
F5 *	30	19.0	±0.5	14	±2	500	127	18.1	±0.8	50	±3	20.7	±1.0	20.4	±0.8	18.7	±0.9
F6	30	13.7	±0.5	18	±2	400	122	12.3	±0.7	61	±4	14.8	±1.0	14.3	±0.7	12.3	±0.7
F7	32	16.5	±0.5	15	±2	400	131	16.1	±0.8	54	±3	19.1	±1.3	18.0	±0.7	16.4	±0.8
F9 *	-	-				2400	558	23.4	±0.4	35	±1	25.9	±0.6	23.5	±0.3	22.8	±0.3
F10	36	22.9	±0.5	11	±1	400	84	21.5	±1.4	60	±5	25.1	±1.5	26.6	±1.6	23.1	±1.6
F11	32	24.5	±0.6	13	±2	400	99	25.2	±1.2	47	±4	28.2	±1.5	29.8	±1.6	26.9	±1.4
F12	29	23.4	±0.7	16	±2	400	88	25.5	±1.3	46	±4	28.5	±1.3	28.7	±1.2	26.6	±1.4
F13	29	22.6	±0.7	17	±2	200	50	21.5	±1.8	60	±6	25.4	±2.1	24.8	±1.7	22.5	±1.9

N: number of aliquots or grains measured; n: number of grains kept; OD: Overdispersion, baSAR-Normal: Bayesian model with a normal (gaussian) distribution; baSAR-LogNormal_M: Bayesian model with a log-normal distribution and median search. We give here the Bayes estimator. In order to facilitate comparisons, the error associated with the Bayes estimator is calculated to 68% from the credibility interval, even though the Bayes estimator is not strictly in the middle of this interval. The samples with an asterix are those processed by Lebrun *et al*. (2016) [22] and mentioned here as a reminder.

Macroscopic and micro-morphological observations performed on thin sections suggest that the sediments were affected by bioturbation, implying a mix of grains of variable ages: there are numerous biological porosities present in samples F1, F3, F4, F6, and F7; dejections in sample F3; termite chambers in proximity to samples F6, F7, and F10. The micro-morphological observations indicate in addition that alluvial and colluvial depositional modes dominate at Fatandi V, which could imply that the grains have been poorly bleached (e.g. [58]). Finally, the De distributions are characterized for some samples by significant overdispersion values (OD) [53]—up to 61 ± 4%—while the OD for the dose recovery tests do not exceed 7 ± 1%. These large OD values could also be due in part to micro-dosimetric factors. In the absence of any precise data with regards to this point it is difficult to come to a conclusion on these different influences and thus to choose the most appropriate age model, if one exists among those considered here.

The absence of sufficiently strong arguments for choosing an age model can be countered in part by the use of Bayesian models that leverage the relative chronology of stratified deposits: grains coming from foreign populations into the population under consideration are somewhat deviant since their apparent age is incompatible with that of the grains located stratigraphically above or below and which are respectively younger or older. Nevertheless, we cannot exclude the possibility that this same process also excludes some grains belonging to the population of interest, but whose apparent ages result from the use of dose rates that are not well-adapted to them. The R package “BayLum” [32,33] (see [30,31] for the mathematical models) allows for the modelling of the OSL sediment sample chronology, taking into account the chrono-stratigraphical order. In addition, the package appropriately manages the errors by taking into consideration those which are common to multiple samples. The R package “ArchaeoPhases” (S1 File) [59,60], also based on Bayesian models, allows respectively the estimation of time intervals for all samples—here the sedimentary units identified at the site.

So as to be able to follow the impact of different statistical models on the estimation of equivalent doses, we first applied the baSAR-Normal and baSAR-LogNormal_M models to the single-grain data without taking into account the stratigraphic constraints (Table 4 and S3 Fig). In the interests of comparison, the arithmetic mean and the CAM (log) were also applied. For all samples except F9, we note that the De of the baSAR-Normal models and the arithmetic mean on the one hand, and the baSAR-LogNormal_M and CAM on the other, are consistent with each other at 1 sigma. However, the De of the pair of statistical models based on the normal laws are 1 to 24% higher than those based on log-normal laws. We see here then that it is the distribution model (normal vs log-normal) rather than the statistical method (Bayesian vs frequentist) which affects the final equivalent dose. This divergence is all the more marked as the distributions become more dispersed (e.g. significant overdispersion). This divergence could simply reflect the deviation between the mean (arithmetic) and the median of the distributions of De [61]. For what here follows, we base ourselves on the work of Guérin *et al*. [61–63] and of Heydari and Guérin [64], suggesting that even—and above all—in the case where the distributions of De are not normal, the baSAR-Normal model is more accurate and more robust than the CAM or the baSAR-LogNormal_M model.

Then the ages were calculated from the single-grain data with the baSAR-Normal model by applying the stratigraphic constraints. These are based on the log presented in Fig 2: F2<F1 (i.e. F2 is younger than F1); F4<F3; F10<F9, F7<F6<F5<F13<F12; F12 is younger than all the samples of U1.

### Results and interpretation of ages

**Fig 10:**
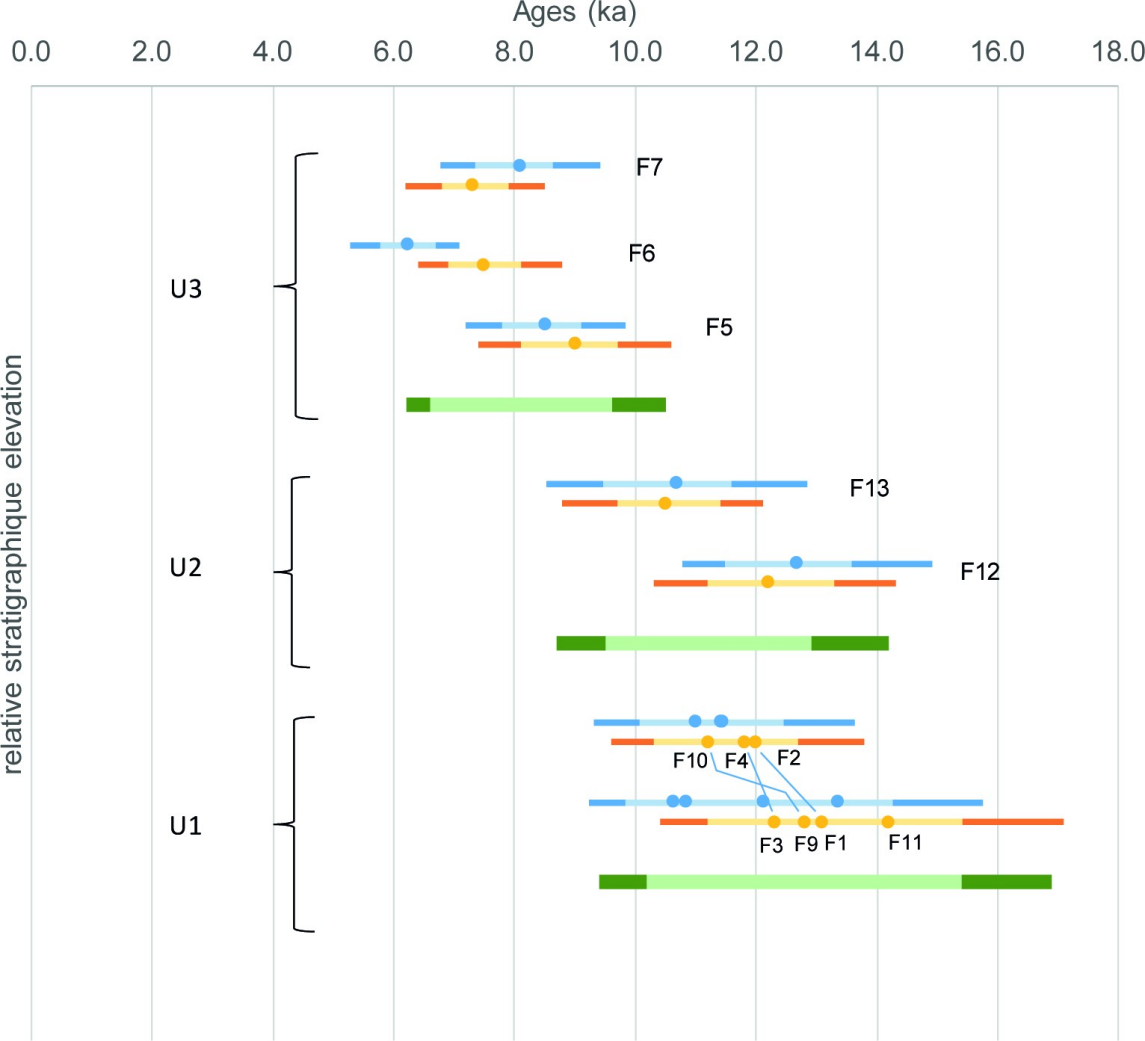
Synthesis of OSL dates obtained with the Bayesian baSAR-Normal model, with (in orange) and without (in blue) stratigraphic constraints. The results of Archeophases for each stratigraphic unit are also indicated (in green). Light and dark colors indicate respectively 68% and 95% confidence level. The relative stratigraphic positions are represented. The chrono-stratigraphic positions of F2, F4, F10 on the one hand and F1, F3, F9, F11 on the other, are not strictly known, hence their representation on the same level. The lines between F1/F2, F3/F4 and F9/F10 remind the relative stratigraphic correlations that are known between the two groups and that have been used in the Bayesian model.

Table 5 and Fig 10 synthesize the ages calculated with the baSAR-Normal model, taking into account the stratigraphic constraints. In the interests of comparison, the ages calculated without any stratigraphic constraints are also indicated. The principal effect of applying the stratigraphic constraints is the resolution of the apparent chrono-stratigraphic inversion between F6 and F7.

**Table 5:** Ages obtained for the single-grain data with the Bayesian baSAR-Normal model, with and without stratigraphic constraints (SC).

Sample	Ages (ka)									
	SG-BaSAR-Normal, no SC					SG BaSAR-Normal SC				
	lower bound at 95%	lower bound at 68%	Bayes estimate	upper bound at 68%	upper bound at 95%	lower bound at 95%	lower bound at 68%	Bayes estimate	upper bound at 68%	upper bound at 95%
F1	10.3	11.0	**12.1**	12.8	14.0	11.0	11.9	**13.1**	14.2	15.4
F2	9.4	10.1	**11.0**	11.7	12.7	10.1	10.9	**12.0**	12.7	13.8
F3	9.2	9.8	**10.6**	11.3	12.0	10.4	11.2	**12.3**	13.1	14.4
F4	9.9	10.5	**11.4**	12.3	13.3	10.2	10.8	**11.8**	12.6	13.6
F5	7.2	7.8	**8.5**	9.1	9.8	7.4	8.1	**9.0**	9.7	10.6
F6	5.3	5.8	**6.2**	6.7	7.1	6.4	6.9	**7.5**	8.1	8.8
F7	6.8	7.4	**8.1**	8.6	9.4	6.2	6.8	**7.3**	7.9	8.5
F9	9.5	10.0	**10.9**	11.5	12.6	10.7	11.6	**12.8**	13.8	15.1
F10	9.3	10.4	**11.4**	12.5	13.6	9.6	10.3	**11.2**	11.9	12.8
F11	11.0	11.9	**13.4**	14.3	15.8	11.6	12.6	**14.2**	15.4	17.1
F12	10.8	11.5	**12.7**	13.6	14.9	10.3	11.2	**12.2**	13.3	14.3
F13	8.5	9.5	**10.7**	11.6	12.9	8.8	9.7	**10.5**	11.4	12.1

The credibility interval boundaries of 68% and 95% are presented as well as the Bayes estimator.

The deposits of the three sedimentary units identified as U1, U2, and U3 would therefore be chronologically associated with the end of MIS2 and the Holocene. The application of the ArchaeoPhases package allows the estimation of intervals at 68% confidence level from 13,400 to 8,200 BCE [15.4 to 10.2 ka at 68%, and 16.9 to 9.4 ka at 95%] for U1, and from 10,900 to 7,500 BCE [12.9 to 9.5 ka at 68%, and 14.2 to 8.7 ka at 95%] for U2, which covers the *in situ* archaeological layer, and from 7,600 to 4,600 BCE [9.6 to 6.6 ka at 68%, and 10.5 to 6.2 ka at 95%] for U3. The F12 sample coming from the *in situ* archaeological layer in the northern part of the site gives an age interval of 11,300–9,200 BCE at 68% confidence level [13.3 to 11.2 ka at 68%, and 14.3 to 10.3 ka at 95%] and places the human occupation on the Pleistocene/Holocene transition. It is also of interest to remember that the F10 sample alone, located 10 cm below the North Square concentration, gives an earlier chronological boundary at 10,800 BCE [12.8 ka] at 95% confidence level. Thus, considering F10 and F12 as framing the layer, a hypothesis to be considered with great caution is a date of the archaeological level between 10,800 and 8,300 BCE [F10: 12.8 ka at 95%; F12: 10.3 ka at 95%], or even between 9,900 and 9,200 BCE with a 68% confidence level [F10: 11.9 ka at 68%; F12: 11.2 ka at 68%]. This would make it possible to attribute the occupation to the Early Holocene. However, considering the uncertainties associated with the use of a single date (without Archeophases) and a 68% confidence level, we will remain cautious in maintaining a chronological placement of the human occupation around the Pleistocene/Holocene boundary.

## Palaeoenvironment results

### Micromorphology and pedology results

**Fig 11:**
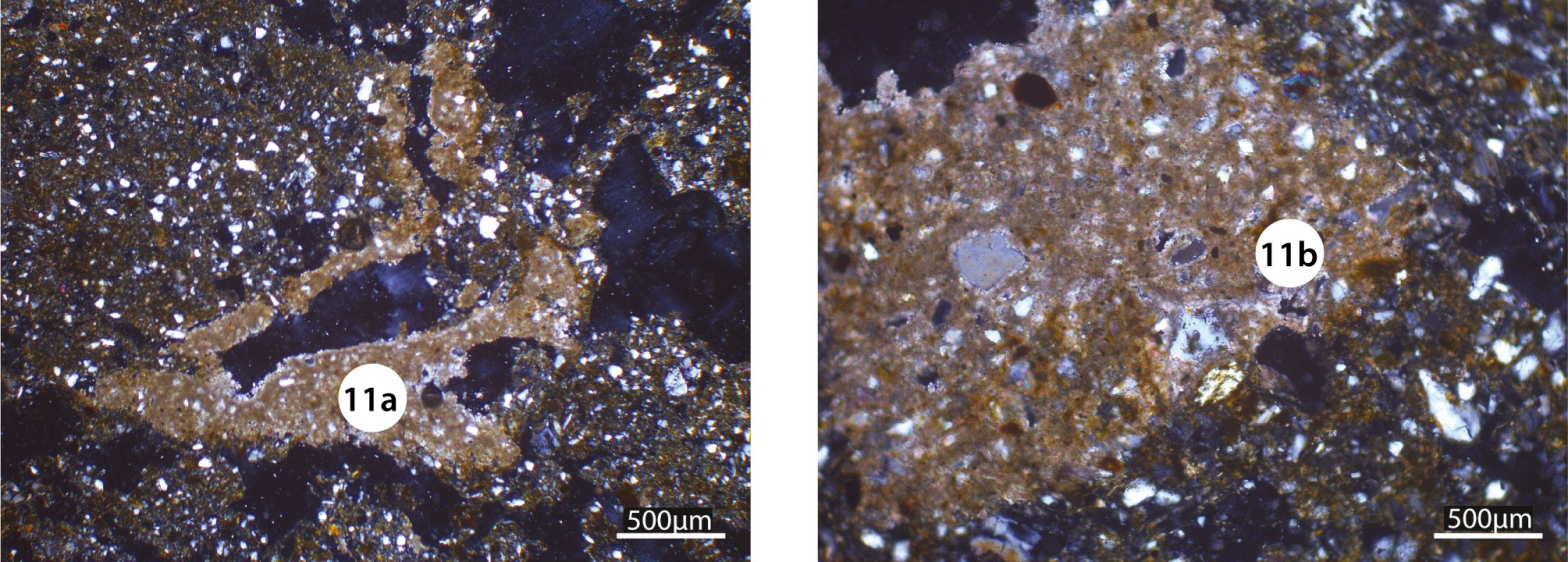
Microphotographs (XPL) of F9 thin section showing calcitic infillings (11a) quasi-coating in a channel structure and a calcitic nodule (11b).

The archaeological level is located above a sedimentary unit corresponding to the silty deposits attributed to the end of Pleistocene and the beginning of the Holocene (U1: 13,400–8,200 BCE [15.4–10.2 ka at 68%, 16.9–9.4 ka at 95%]) (Fig 2). The micro-morphological examination shows that the basal mass corresponds to the quartzite silts (20μm) comprising between 15 and 20% fine sands (100–200μm). The fine sediments are partly wind-blown in origin though, as indicated by their concentration in fine sands, they must have been redistributed on light slopes by runoff and by the action of the Falémé during periods of flood. These deposits include numerous pedological traits that suggest they underwent post-depositional pedogenesis. This is underlined by a macroporosity that structures the sediment in angular to sub-angular aggregates. This has however partially collapsed (F2). The thin sections testify to the strong development of channels and chambers that indicate a porosity of biological origin, though the most remarkable is the presence of carbonate characteristics which allow the bottom of the pedological profile to be identified. Most of the voids are characterized by clay alluvial coatings. At the bottom, it can be observed that the voids possess a whole or partial alluvial coating and that numerous calcitic nodules are integrated into the matrix (F3, F9, Fig 11). At the top, the thin sections F4 and F2 present two generations of clay revetments. The first corresponds to hyaline clays, which are relatively pure in comparison to the second, which are often dustier. Together, these pedological characteristics indicate an accumulation horizon of fersiallitic soils. In contrast to today’s iron-rich soils characteristic of the Sudanic zone and of the region studied [65], this indicates an incomplete carbonate washing, which suggests a climate with seasonal contrasts but within the cooler climatic conditions one finds today north of the Sahara in the Mediterranean world. It is the surface of these soils that were occupied by the populations of Fatandi right at Pleistocene/Holocene boundary. The sign of their occupation may be seen in the truncated pedological profile, which is missing the surface and eluvial horizons. This degradation of the surface horizons also explains the second generation of dusty illuviations in the voids within the upper part of the palaeosoil accumulation horizon. This phase probably indicates the low seasonal level of the Falémé, which would have allowed the establishment of populations on the alluvial plain or on an alluvial terrace under construction, as well as the erosion of soils by seasonal rains.

This erosion explains the establishment of a second sedimentary unit (U2: 10,900–7,500 BCE [12.9–9.5 ka at 68%, 14.2–8.7 ka at 95%]). This corresponds to silty-sandy sediments containing or covering archaeological artefacts (Fig 2). These sediments are composed of thin colluvial layers that accumulated along the gentle slope of a hill and came from the accumulation eroded out of the fersiallitic soil, linked to the occupation of the site of Fatandi. They are thus contemporary to the occupation and are distinguished by a later pedogenesis. This is characterized by a granular to subangular blocky structure and several clayey coatings, as well as significant rubefication of the groundmass. This indicates the return of pedogenesis during warmer and more humid conditions, as is also suggested by the absence of calcitic traits and the mobility of iron.

### Vegetation

**Fig 12:**
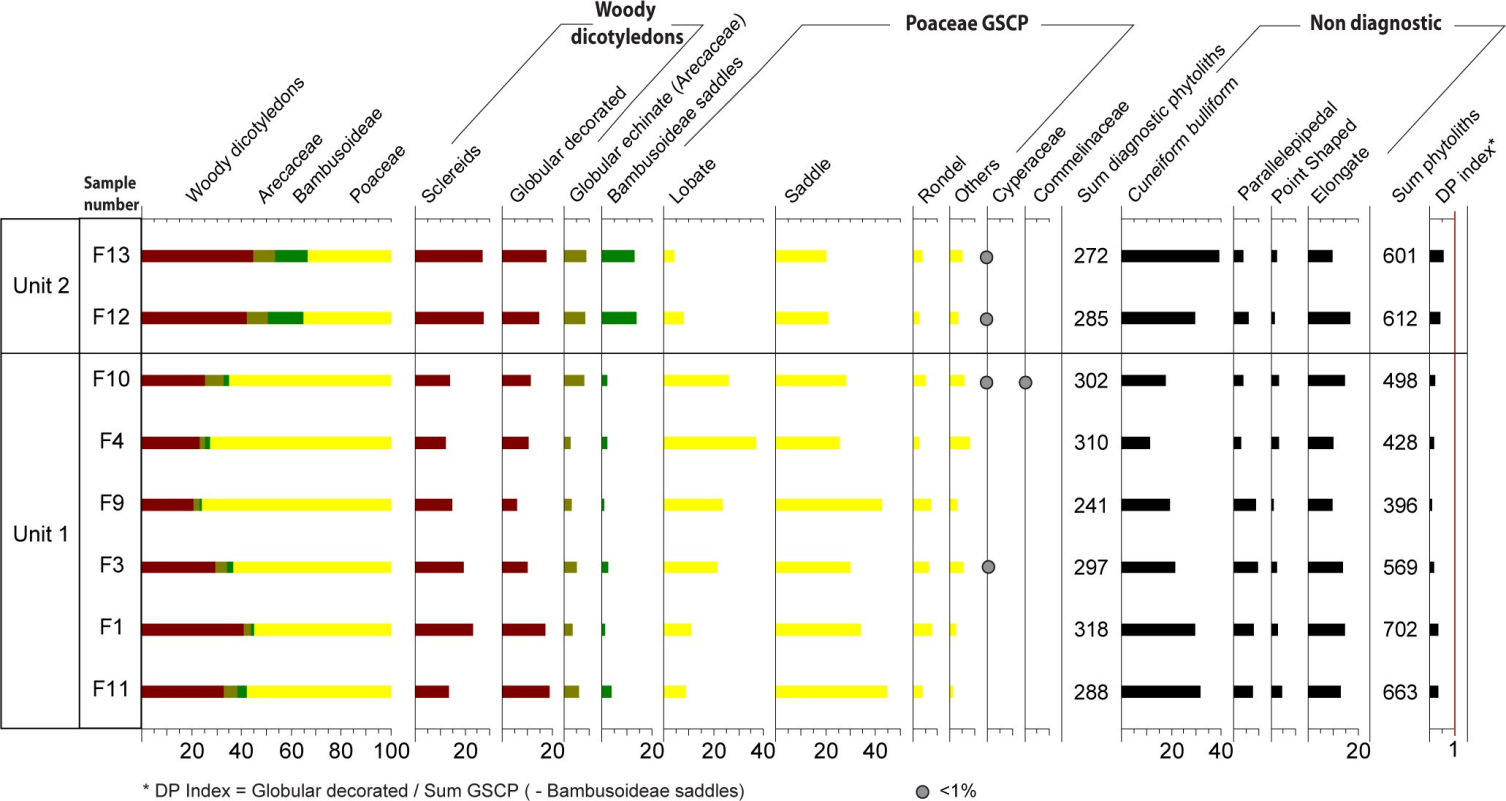
Phytoliths assemblages of eight samples from U1 and U2 in Fatandi V. The stratigraphic relationships between the U1 samples have not been indicated here to better highlight the links between F1 and F11 on the one hand and between F3, F4, F9 and F10 on the other hand.

Phytolithic analysis has been conducted on the sediments from samples F1, F3, F4, F9, F10 and F11 collected from U1, and F12 and F13 coming from U2. The phytoliths assemblages of the samples F11 and F1 from U1 (13400–8200 BCE [15.4–10.2 ka at 68%, 16.9–9.4 ka at 95%]) are similar and testify to a vegetation dominated by short grass layer with sparse woody plants (Fig 12). GSCP (Grass Short Cell Phytoliths) are well represented reaching 55–58% but are strongly dominated by saddles morphotypes (33–44%). Saddles are mainly produced by Chloridoideae, and further suggest the prevalence of a warm and dry climate. Woody dicotyledons morphotypes record values of 32–40% and suggest the development of a ligneous tree and shrub stratum. Arecaceae morphotypes represent 5 and 3%. Samples F3, F4, F9 and F10 indicate a more open vegetation. GSCP strongly increase with values of 63–76% while woody dicotyledon phytoliths (Sclereids and globulars) are present in lesser proportions (20–29%). The better representation of lobate GSCP (22–37%) associated to Panicoideae sub-family suggest a more humid grass cover. Moreover, the presence of *Commelina forskaolii* [66] and Cyperaceae morphotypes in the F10 assemblage testify also to more humid conditions.

The samples F12 and F13 from the Unit 2 (10,900–7,500 BCE [12.9–9.5 ka at 68%, 14.2–8.7 ka at 95%]) testify to a more closed vegetation cover (Fig 12). Woody dicotyledons morphotypes increase reaching 42% and 44% and globular echinate (Arecaceae) are more abundant (8%). The most conspicuous feature is the high percentage of Bambusoideae GSCP, with values of 13% and 14% respectively. These assemblages attest of the development of a humid gallery forest.

As regards samples from U1, it is difficult to distinguish them chronologically. Although phytolithic differences appear among the samples of U1 and it is then possible to discuss their succession, it is above all the very clear evolution of the landscape between U1 and U2 that we will retain. The environmental ambience related to the Holocene perfectly supports the idea of a human occupation dated around the Pleistocene/Holocene boundary and the return of more humid and warm conditions.

The stratigraphic position of the archaeological level at the interface of two sedimentary units affected by erosion and accumulation phenomena as well as the vegetation cover context confirm the interest of a taphonomic analysis to better understand the archaeological deposition processes in this environmental transition framework.

## Taphonomy

### Stratigraphic data

In the Concentration 1, artefacts were completely or near-completely eroded and lying on the surface. Only a few remaining objects are in stratigraphy within the first 10 centimeters of the sediments: these represent either artefacts that have undergone vertical migration, or maybe the last objects still deposited *in situ*. Beyond these 10 centimeters, the below sediments are sterile. The edges of artefacts are fresh, but some are highly weathered and patinated. It could suggest either an archaeological mix or a mixture of artefacts of different origin from the main occupation.

In the Concentration 2, two sub-concentrations were being eroded, but almost 500 artefacts were still in a well stratified archaeological level (Fig 5). The below sediments are sterile. This sector therefore seems to testify to a single occupation (or a single level with palimpsests of occupation) that has been significantly eroded.

In the North trench and its extension, the stratified artefacts are also within one single archaeological layer of some 10 cm thick (Fig 5). Some southern sections of the North trench present either surface artefacts (small concentrations which have been completely eroded) or a mix of stratified and reworked artefacts, as in the Concentration 2, thus supporting the model of an eroded single layer.

In the North square, the existence of a clearly *in situ* occupation was confirmed by the discovery of dense concentration of more than 700 artefacts, measuring roughly 1m^2^ by 10 cm thickness, preserved beneath 30 cm of sediments (Figs 3D, 5 and 6). The high density of the artefacts compared to the other areas of the site and the thinness of the level in stratigraphy support the idea of an *in situ* archaeological layer.

In summary, the most northern excavated areas only present artefacts still in stratigraphy. It is nonetheless necessary to distinguish between artefacts located in Concentration 2 and the North square on the one hand, and those recorded in the North trench extension on the other hand. Lithics from Concentration 2 and North square are stratigraphically located at the interface of U1 and U2, and therefore should be considered part of an *in situ* layer, deposited on a U1 soil (Fig 2). Undisturbed or very slightly moved from their original location, these artefacts were then buried by U2 sediments. As regards the northernmost artefacts, these were located within U2 (Fig 2). The low density of lithics in this area and the absence of a clear eroding limit between U1 and U2 supports that these artefacts were disturbed from the U1/U2 interface due to an erosion of the archaeological layer and subsequently deposited in an episode of colluviation, following the slope at that time, probably not far from their point of origin.

### Analysis of distribution of Concentration 1 artefacts

**Fig 13:**
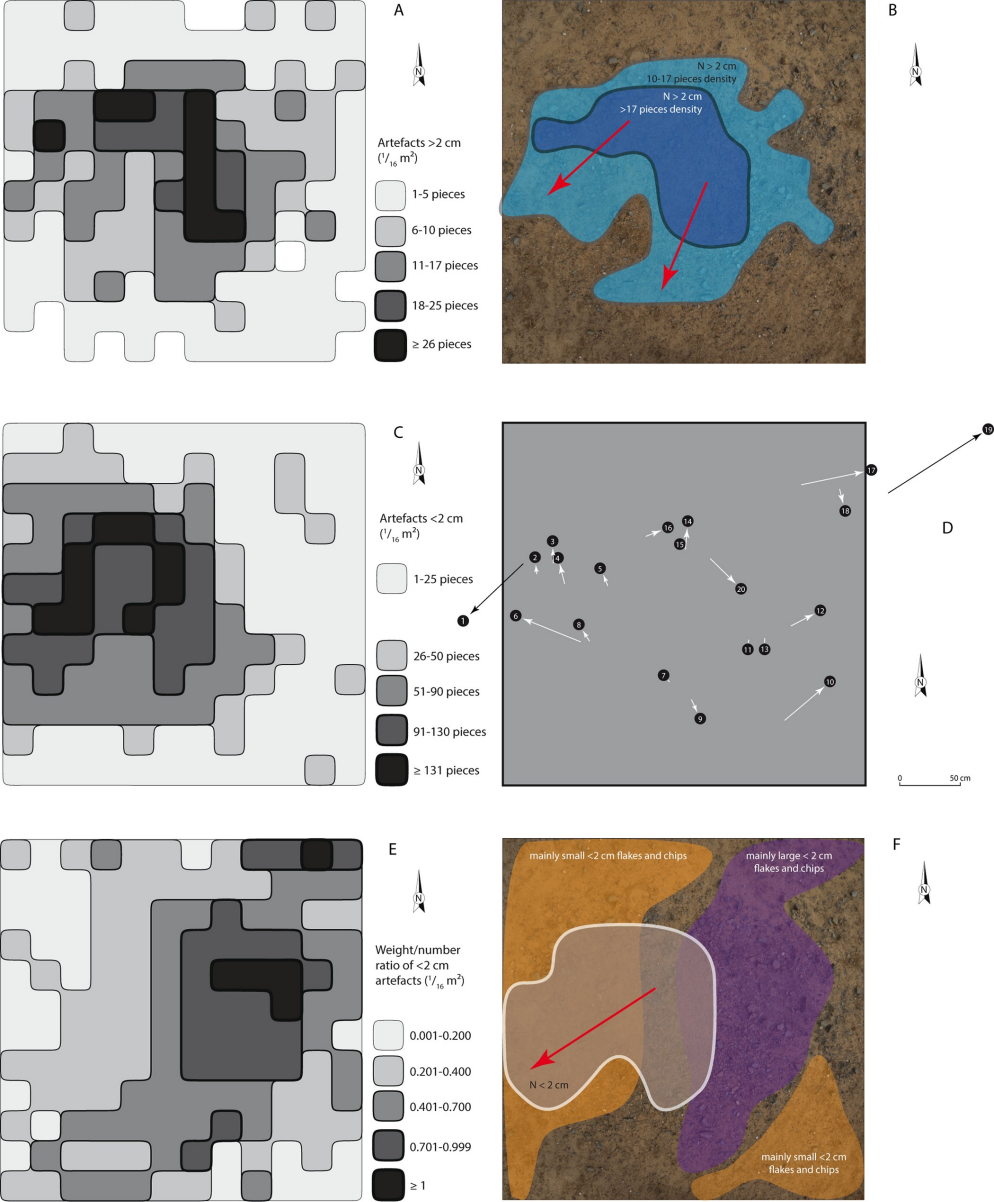
Quantitative data per ^1^/_16_ m^2^ and spatial interpretation of the distribution of Concentration 1 artefacts. (A) Distribution of the artefacts longer than 2 cm. (B) Spatial interpretation of the concentration and diffusion of the artefacts longer than 2 cm. (C) Distribution of the artefacts smaller than 2 cm. (D) Displacement of 20 localized artefacts between 2012 and 2013 field missions. (E) Distribution of the weight/number ratio of the artefacts smaller than 2 cm. (F) Spatial interpretation of the concentration and diffusion of the artefacts smaller than 2 cm.

The quality and quantity of finds from Concentration 1 allowed us to analyze the dispersion process of eroded artefacts by studying their current distribution. The analysis shows that the 1,180 pieces longer than 2 cm are mostly located at the center of the concentration, with a distinct diffusion towards the west (Fig 13A and 13B). The large number of artefacts smaller than 2 cm (N = 7,348) are more widely distributed, with the largest amount in the western part of the concentration (Fig 13C). For this same dimensional category, the ratio between the weight and the number of collected artefacts allows us to distinguish three zones (Fig 13E and 13F):

- A western zone, predominantly composed of the smallest elements
- A south-eastern zone, with the same type of elements
- An eastern and north-eastern zone, where small lithic fragments are larger.

Measuring the surface altitude of the area after collecting artefacts indicates that the center is slightly more elevated (6 cm), with a slight slope towards the south-west, similar to that of the site. The distribution is therefore logically from the centre to the periphery, accentuated by the topography of the area or by the differential erosion of fine sediments between the center and the periphery. In particular for the smallest elements, the spread towards the west and south-west may be explained by the winter rains and to a lesser extent by trampling.

In parallel, 20 artefacts identified in 2012 were re-located in 2013. The 20 artefacts together present a movement that is greater for those artefacts that are farther from the center of the concentration. The displacement varies between approximately 10 cm and 1 m (Fig 13D). The surface run-off of the rainy season as well as animal and human trampling can easily explain these movements.

It can therefore be seen that the original distribution is on the whole preserved, albeit with a relatively more significant dispersion towards the edges and more disturbance for these peripheral artefacts.

**Fig 14:**
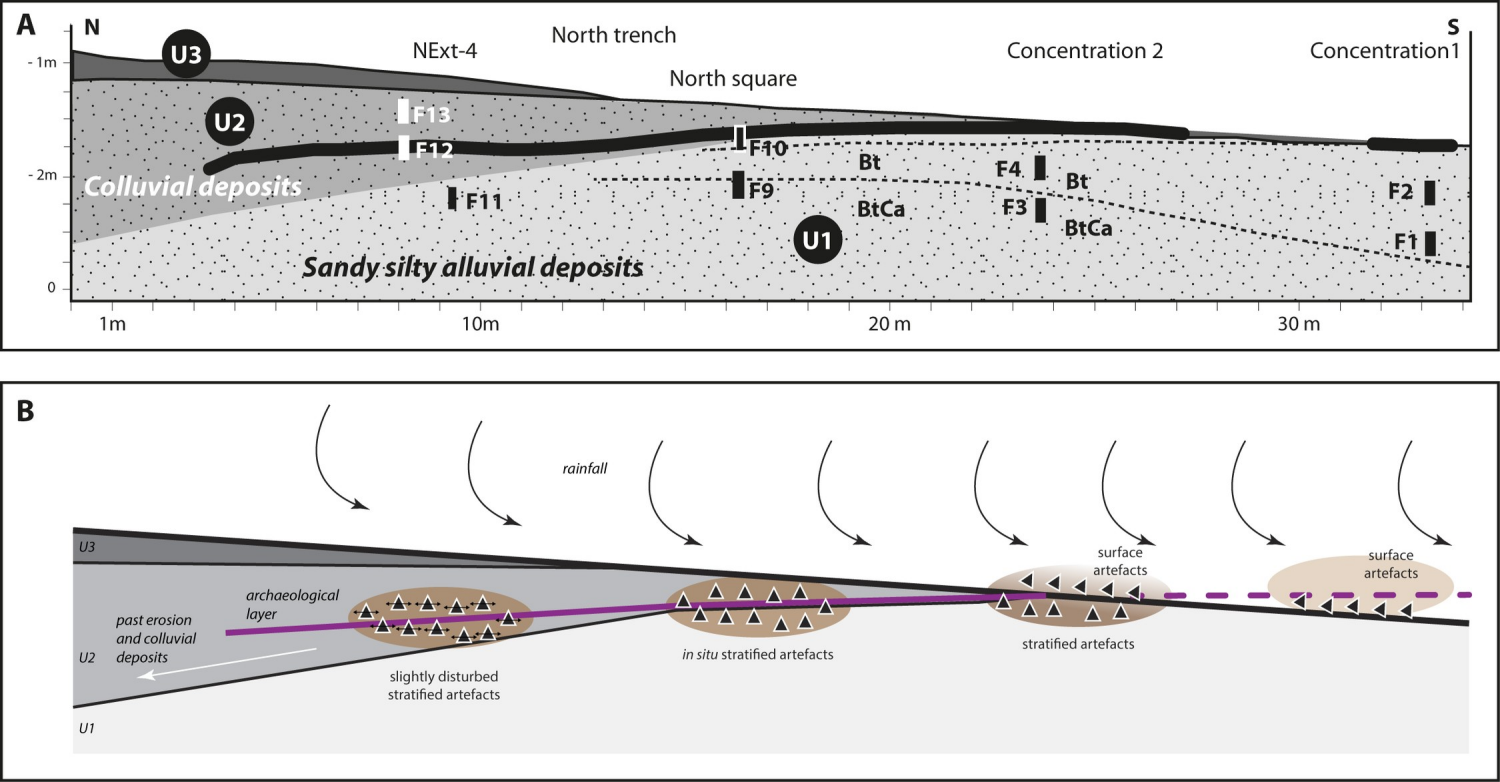
Interpretative model of the erosion of the archaeological level (B) compared to geomorphological and stratigraphical data (A).

Fatandi V thus probably shows a single archaeological occupation (with several potentially more recent elements on the surface), at multiple stages of erosion, represented by (Fig 14):

- Sector Concentration 1 and the South trench, eroded
- Sector Concentration 2 and the southern half of the North trench, in progress of erosion
- The North square and the northern part of the North trench, with the *in situ* layer preserved beneath 30 cm of sediments
- The extension of the North trench, in which artefacts were found in stratigraphy beneath 70 cm of sediments, but likely slightly disturbed.

Although the possibility of palimpsests of several mixed archaeological levels on the surface in the Concentration 1 area cannot be ruled out, the hypothesis of a single level throughout the site is proposed. The hypothesis of palimpsests of occupation by one or more groups within this level is obviously possible.

Together, these observations demonstrate the past and current erosive dynamics. The archaeological layer extends northwards on a slope slightly inverse to that of today, and runs therefore between 60 and 70 cm under the surface of section NExt-4. The occupation thus appears in a primary context between Concentration 2 and the North square, stratified yet slightly disturbed within the North trench extension, eroded by current and past episodes of precipitation and run off to the south of Concentration 2.

The erosion seems relatively rapid, as suggest those artefacts of Concentration 2 eroded between 2012 and 2013. Once on the surface, the spread of archaeological artefacts is aggravated by rainfall, progressively degrading the glacis, and animal trampling, itself explained by the dense pastoral activity on the banks of the Falémé during the dry season.

It is currently difficult to give a functional interpretation of these concentrations. It is nonetheless worth noting the significant density of the concentration of the North square: more than 700 artefacts for an area less than 1 m^2^. Outside of this area, this distribution very quickly becomes sparse, if not completely sterile. Beyond the classic interpretation as a knapping pile, it is also possible to interpret it as a refuse mound, removed from the knapping zone. An analysis of the state of preservation of the concentration, in particular of its micro-stratigraphy, could shed some light on this issue.

## Lithic analysis

### General typo-technological count

**Fig 15:**
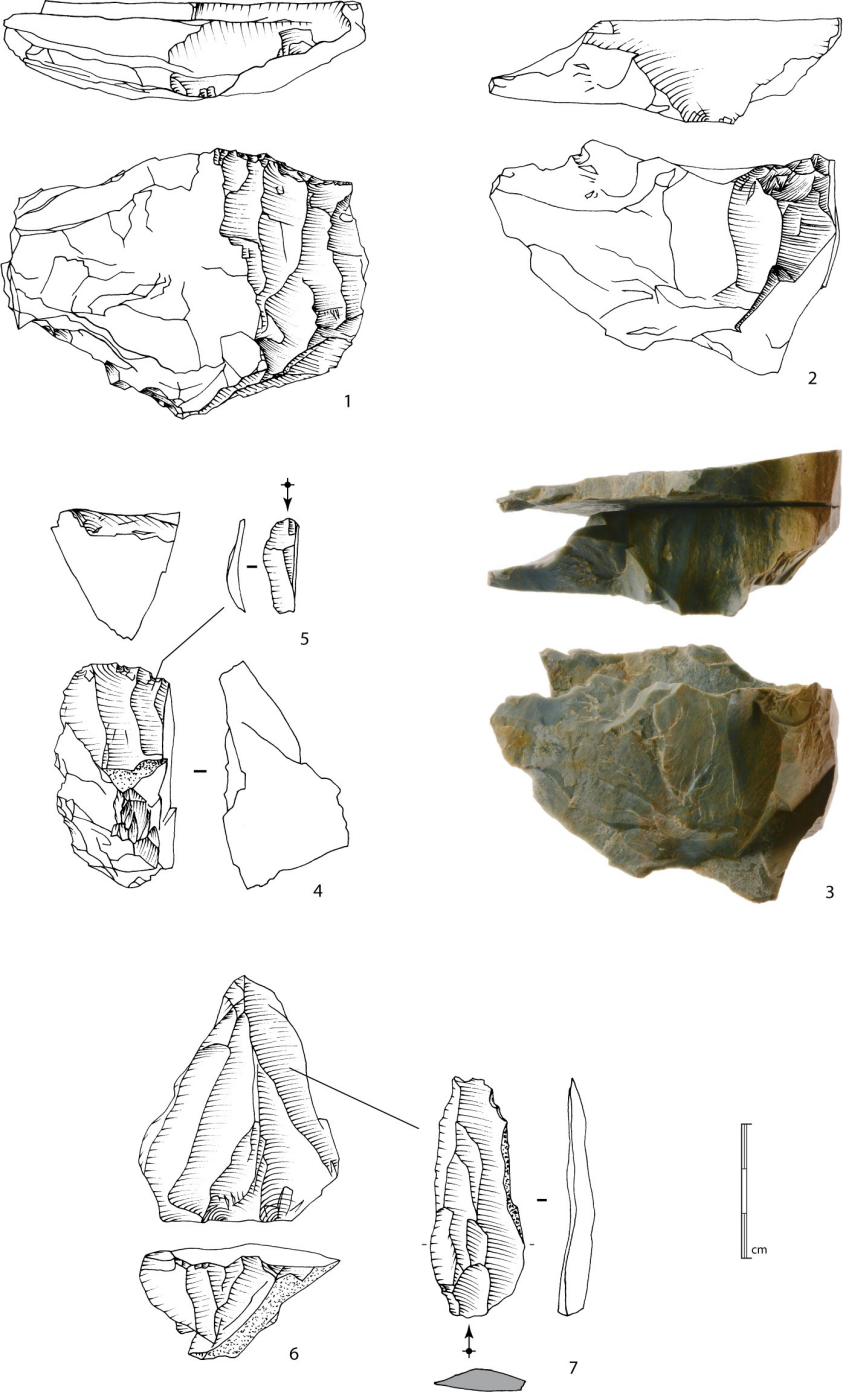
Bladelet production. 1 to 3: Method 1 bladelet cores; note the refitting of a splitted slab on n°3. 4: Bladelet core with frontal exploitation of a ridge. 5: Narrow bladelet refitted on the n°4 bladelet core. 6: Bladelet core with convergent production organized following two asymmetric surfaces. 7: *Debordant* short blade refitted on the n°6 bladelet core (drawings and photographs B. Chevrier).

The North square and its extensions produced 729 lithic artefacts longer than 2 cm, all in a very fresh state of preservation. 406 artefacts, or 55.7%, present confirmed traces of knapping, 205 artefacts (28.1%) with uncertain knapping indication and 118 (16.2%) are natural pieces. The uncertainty of human intervention and the high proportion of natural pieces is due in particular to the significant fissuring of the blocks, which could have exploded or split during knapping activity (Fig 15, n°3).

The 406 artefacts with confirmed knapping scars consist of cores and negative bases, flakes, bladelets and blades. The negative bases represent blanks with flake scars, but from which it is impossible to state on the true intention of flaking or shaping [45]. The toolkit is composed of segments and natural artefacts with confirmed or plausibly retouched edge. Waste, undetermined pieces and hammerstone fragments complete the assemblage (Table 6).

**Table 6:** Inventory of the lithic assemblage from North square of Fatandi V.

Nature of pieces	N	%
Core	23	3.2
Negative base	17	2.3
Flake	257	35.3
Bladelet	38	5.2
Blade	8	1.1
Segment	1	0.1
Retouched natural blank	9	1.2
Waste	4	0.5
Hammerstone fragment	4	0.5
Undetermined	45	6.2
Lithics with uncertain knapping scars	205	28.1
Natural pieces	118	16.2
**Total**	**729**	**100.0**

From the North trench, three more cores were analyzed, as well as a flake and a blade with interesting technical information. In addition, a broken hematite pebble showing striations is also documented in this section.

### Procurement of raw materials

With regards to the raw materials, blue-green jaspoid grauwacke is used almost exclusively. This raw material is regionally found in centimetric to decimetric banks located in a silt-clay basement of the schisto-pelitic sedimentary series [67]. Those blocks that have not been weathered and have fresh edges are probably blanks recovered in their primary context in the form of slabs. It is a fine-grained material with excellent knapping properties. Nonetheless, the numerous parallel and orthogonal fissures could prevent an easy and efficient knapping procedure. Many of the artefacts considered to be negative bases are likely the result of the block breaking on impact. The knappers therefore recovered the already fractured slabs from the outcrop or fractured larger blocks during the knapping operations. These cracks would have required cleaning of the block prior to any debitage production in any case and would have impeded the exploitation of larger blocks. Further south, towards Alinguel, outcrops of grauwacke were identified in 2012 within schist or sandstone: we already noted their very fractured state in this context.

A dozen artefacts present a brown color that seems to correspond to a distinct facies, somewhat grainier, within the same raw material.

A segment collected in the North square was made of a whitish-green material, lightly chalcedoneous, whose provenance is unknown. The only other artefact from the layer produced from the same material is another segment from the same type collected in NExt-4/Ext-1: in this case, the object presents a natural brown surface.

No artefact made of quartz or sandstone (other than the hammerstone fragments) were identified. The knappers must therefore have opted exclusively for very fine raw materials with excellent knapping properties, despite the presence of disturbing fissures.

### Modes of debitage and resultant blanks

Since the majority of flakes were not diagnostic and the conceptual process of debitage was relatively simple with little evidence for preparation, we checked the produced blanks and selected several flakes that provided interesting technical information. We mainly focused on the most informative artefacts—cores, bladelets and blades—and secondarily on the negative bases. Three cores from the North trench were added. The technical analysis identified the conceptual processes that governed the two main types of production: bladelet production and flake debitage.

#### Bladelet production

A total of 14 cores show bladelet negatives, to which can be added five blocks whose identification remains uncertain. Several methods can be identified (the *chaînes opératoires* for two blocks remain undetermined). However, the small sample size and the fact that some methods are only recognized as single units mean that we must remain cautious about distinguishing and characterizing them. A more extensive excavation and a larger corpus would make it possible to confirm or qualify these differences.

**Fig 16:**
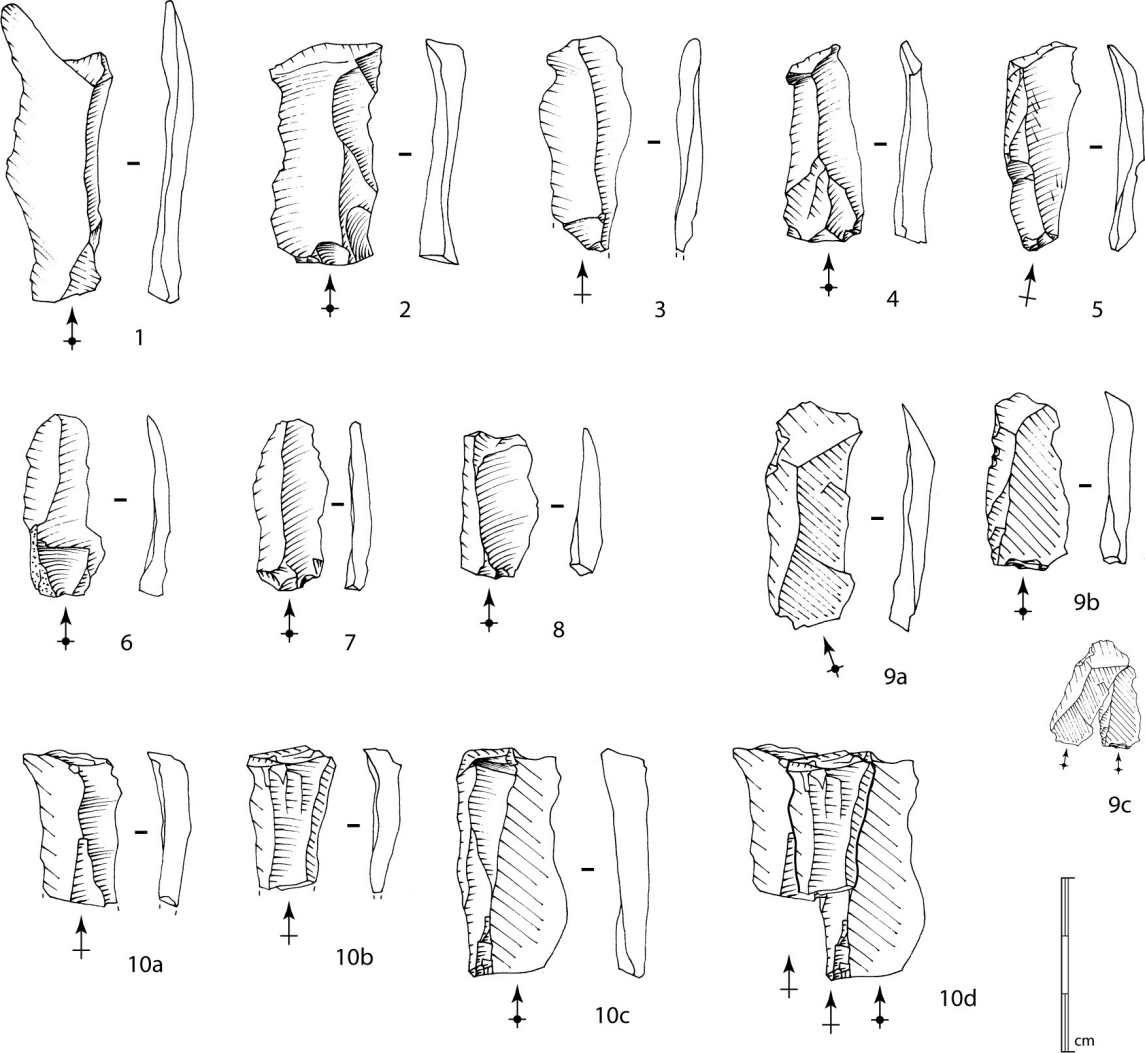
Bladelets. 1 to 8, and 9a to 9b: Bladelets produced from method 1 bladelet cores. 9c: Refitting of 9a and 9b bladelets. 10a to 10c: Bladelets produced from method 2 bladelet core. 10d: Refitting of 10a to 10c bladelets (drawings B. Chevrier).

The first method is identified on 11 blocks (as well as, although less conclusively, on three others) and on two of the three cores from the North trench. This method corresponds to the exploitation of a natural ridge or a lateral crest adjacent to a large surface (Fig 15, n°1 to 3). The production, which occurs obliquely to the main axis of the blank, is either concentrated along this ridge or extends to the wide surface. The bladelet scars seem to indicate a production of straight bladelets, probably quite thin and relatively wide. The scars on cores n°1 and 2 in Fig 15, for example, show a mesial width between approximately 0.9 and 1.2 cm. As the negatives are partly overlapped by more recent ones, it is reasonable to consider that some bladelets should be between 1.5 and 2 cm. This type of blanks has been recognized among the small set of bladelets (Fig 16, n°1 to 8, 9a to 9c). The widest bladelets shown in Fig 16 measure 1.9 cm at their widest point. A quantitative and metric analysis of the scars and the blanks on a larger corpus of cores and bladelets would confirm this first observation. The preparation is limited to the preparation of a striking platform surface on one side of the block by removing one or two flakes, and occasionally the natural dihedral can be knapped by removing a few flakes, forming a unilateral crest. The production surface is usually composed of natural surfaces.

One blank presents possible evidence for the exploitation of a central ridge on a large surface (method 2), as already identified within sector Concentration 1 [9,48]. The refitting of three bladelets also suggests this choice of production method, which supports the idea that a primary aim was to obtain wide bladelets with a rectilinear profile (Fig 16, n°10a to 10d). In this case, preparation is limited to the opening of a striking platform surface, and the exploitation of the block starts on a natural exploitation surface.

For these first two methods, the low level of investment in the cores also appears in the bladelets obtained: natural surfaces on the upper faces (Fig 16, n°6, 9a and 9b), plain striking platforms, rarely dihedral. The production series are limited to a few bladelets before hinge terminations occur, preventing sustained exploitation. The absence of any evidence for maintenance indicate that these episodes of production are limited to the preparation of the block and a limited production, which can be explained by the numerous fissures within the blocks.

One core (and possibly a second) show the frontal exploitation of a ridge (Fig 15, n°4). The core is exploited after the creation of a bilateral crest (partial or total). Here also, apart from the small scars of the crest, preparation is limited to the opening of a striking platform surface. One laminar flake from the North trench also presents frontal exploitation across the width of a block with mainly natural surfaces. The platform is plain and natural. This production method was already recognized on surface artefacts of Concentration 1 [9,48]. The produced bladelets have to be logically narrower than those from the first two methods of production (Fig 15, n°5). They may only form a small part of the assemblage. Their production seems limited to a few removals, and maintenance of the blocks was not observed: the exploitation thus finishes with successive hinge fractures.

One core from the North trench shows a rather unusual method of exploitation: it is organized following two asymmetric surfaces which represent respectively the surface of debitage and the striking surface (Fig 15, n°6). Three or four small convergent removals form the striking surface for the production of convergent bladelets on the debitage surface. This surface is slightly convex, seemingly little prepared, and the refit of a short blade suggests the production of *debordant* elements (Fig 15, n°6–7). It is quite difficult to characterize precisely this kind of production based on a single block. A Levallois conceptual approach of the block is one hypothesis: in order to confirm or reject this, it would be necessary to consider the *debordant* bladelet removals as maintenance flakes, a presumption that remains to be proved. For the moment, in the absence of other artefacts with similar characteristics, we can only postulate that this core shows the exploitation of a slightly convex surface, hierarchical with regard to the striking surface and allowing for the production of wide convergent bladelets, several of which are *debordant*.

#### Flake production

Nine cores present flake negatives, some of which are on the bladelet cores. Two other blocks are considered to be uncertain flake cores. Two methods have been identified.

**Fig 17:**
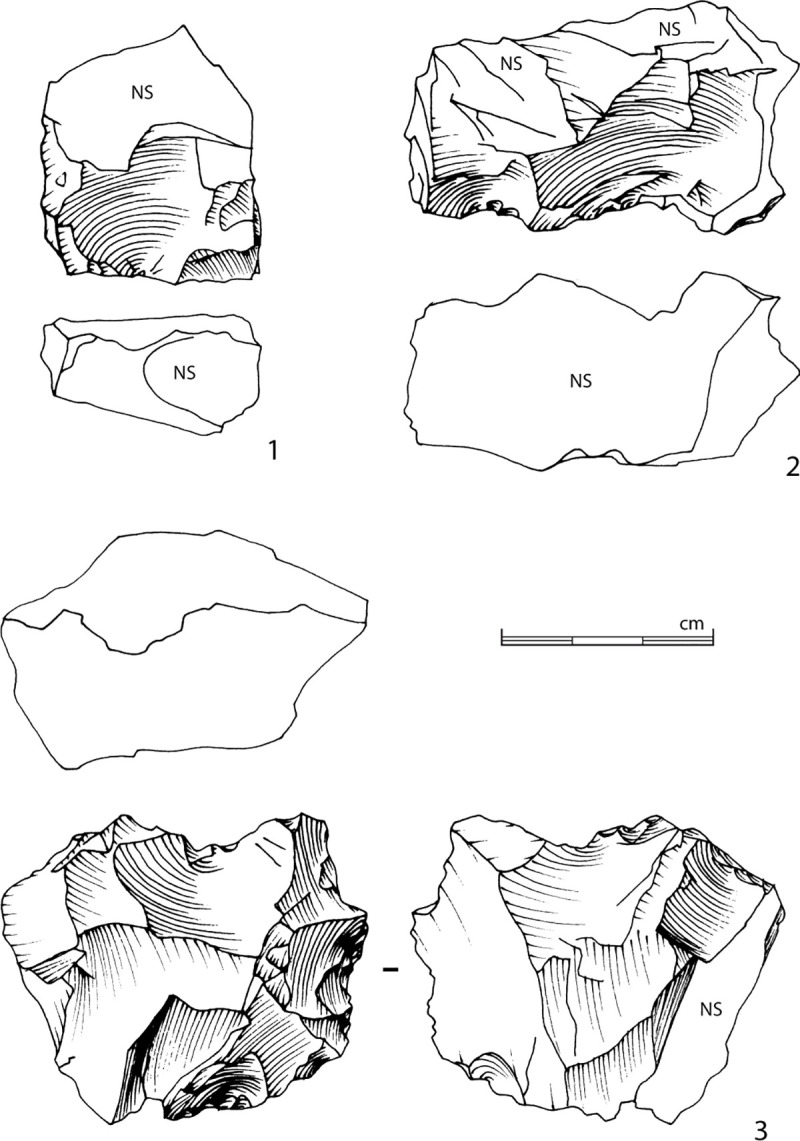
Flake cores. NS = natural surface (drawings B. Chevrier).

The first method represents a very simple production method, based on the search for suitable hinges and surfaces for the removal of short flakes, generally quadrangular in shape. The blocks are generally small in dimension (Fig 17). However, since the largest flake core (Fig 11, n° 3) measures 5.2 cm in the abandoned state with many removal scars, it is difficult to estimate the original size of the core and the size range of the knapped blocks without a larger corpus of flake cores. We can only observe that at least small blocks of the order of a few centimetres have been knapped. Given the strong natural cracking of the blocks, significant exploitation of large blocks larger than 10 cm is unlikely. No preparation of the blocks was noted, even the preparation of a striking platform. Production series are limited to several removals, if not only one. One core presents multiple striking surfaces and a discoidal volume (Fig 17, n°3). Knapping of this block is peripheral and realized on two asymmetric faces, and fewer than ten removals were made.

**Fig 18:**
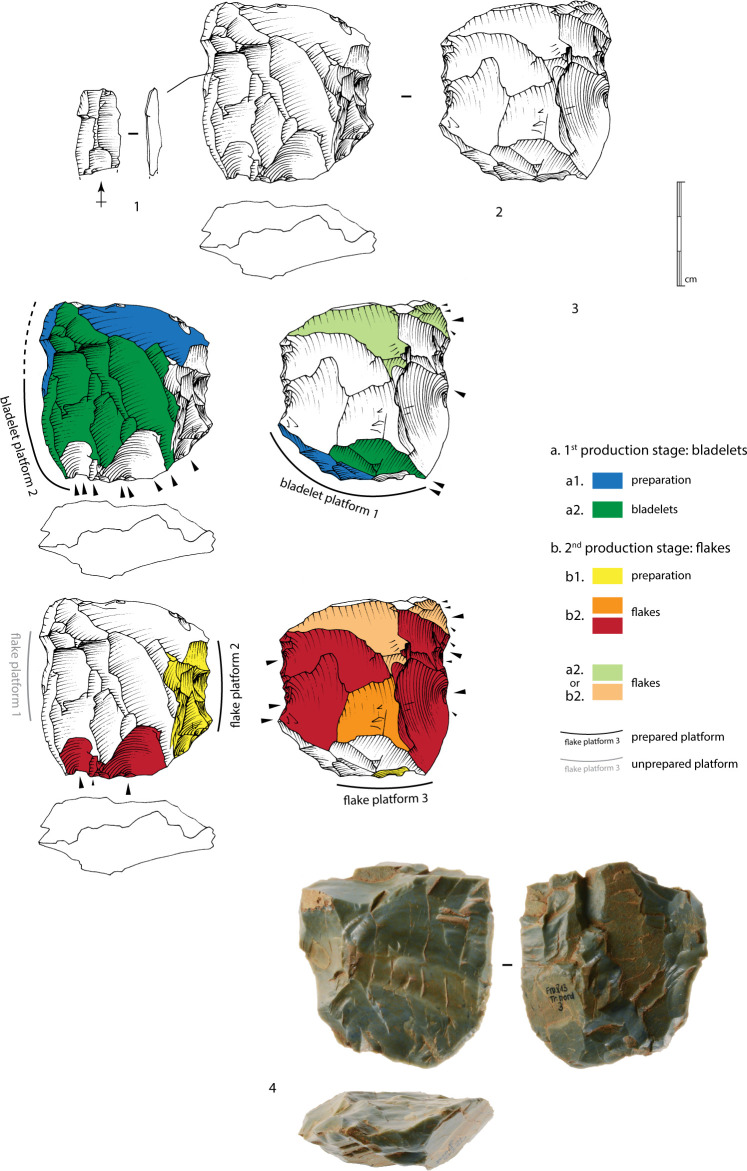
Core from North square. 1: Bladelet refitted on core n°2. 2: Bladelet and flake core. 3: Technological analysis of the *chaîne opératoire* showing two stages of production (bladelet debitage, then flake debitage). 4: Photograph of the core (drawings and photographs B. Chevrier).

One core from the North square and a core from the North trench present flake production, but only following the production of bladelets. The debitage takes advantage of already existing characteristics of the bladelet cores, in particular the convexities on the debitage surface (Fig 18). Where needed, striking platforms were prepared. For one of the cores, short flakes were produced in multiple opposed and orthogonal series. For the other one, an invasive removal as well as a short flake removed almost the entire debitage surface. The volume of these two pieces, composed of two more or less symmetrical surfaces, is reminiscent of a Levallois approach. However, although the necessary surface and convexity criteria are close to those of the Levallois concept, no preparation or management of lateral or distal convexities is apparent. Indeed, the opportunistic nature of these debitages, following the production of bladelets, suggests rather the simple purpose of producing short or longer flakes from suitable surfaces and intersections of striking platforms and exploitation surfaces.

#### Tools

Retouched tools are scarce: only the segment from the North square and eight retouched artefacts (confirmed or nearly confirmed) were analyzed. The few number of retouched pieces may suggest that unretouched bladelets and flakes may have been used as tools, but only a comparative technofunctional and metric study on a large amount of these blanks and a use-wear analysis could provide some results.

**Fig 19:**
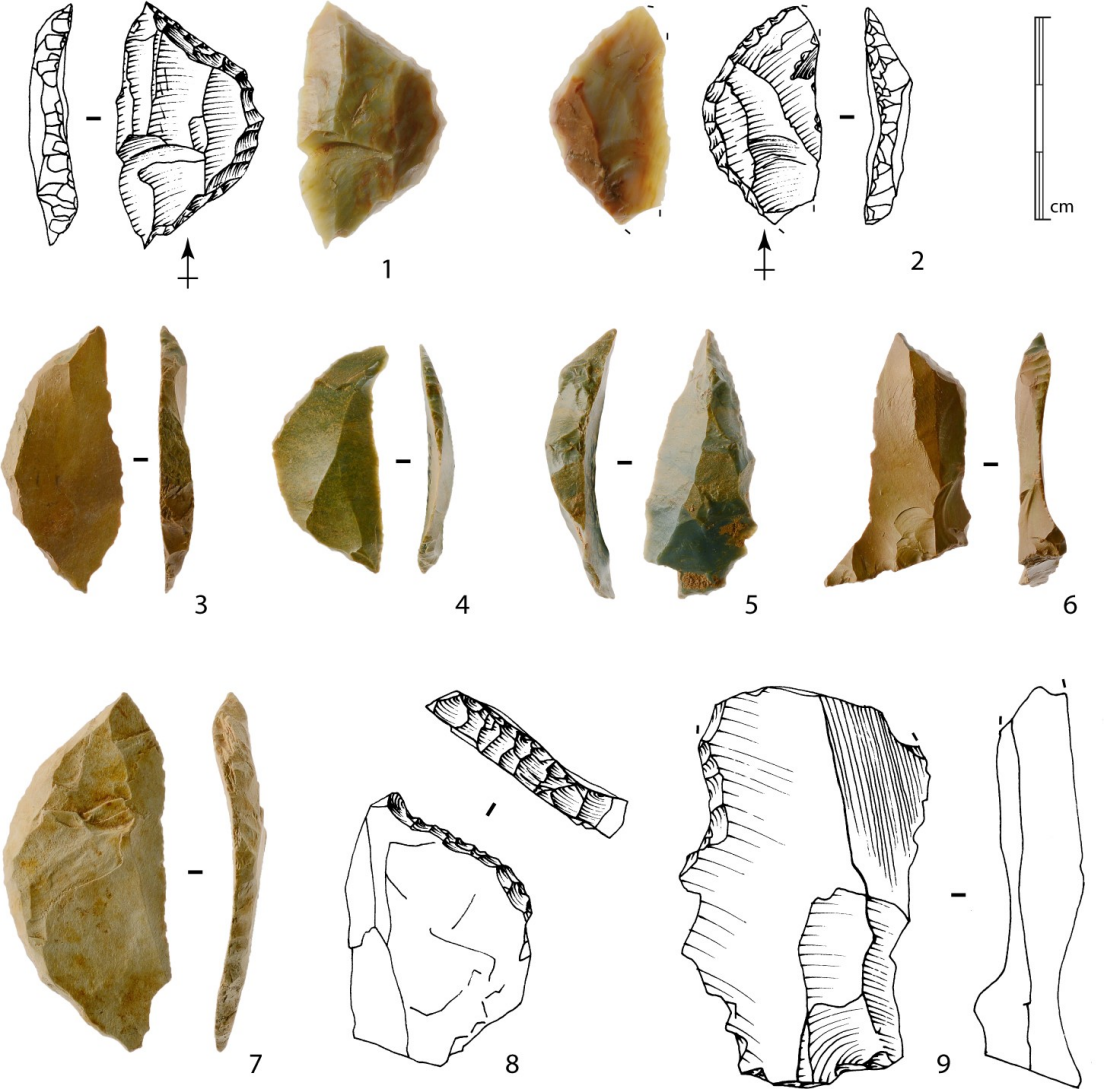
Toolkit from excavations and surface. 1: Segment from North square. 2: Segment from Next-4 section. 3 to 6: Segments collected on surface, close to excavated sectors, made on jaspoidal grauwacke. 7: Segment collected on surface, close to excavated sectors, longer than the other segments and highly weathered. 8: Retouched natural blank. 9: Retouched elongated flake (drawings and photographs B. Chevrier).

As regards retouched artefacts, the segment measures 3.5 cm long, 2.2 cm wide and 0.7 cm thick. It is made of a fine whitish-green, lightly chalcedoneous siliceous material (Fig 19, n°1). It was probably made from an elongated blank, as the upper face presents multiple unidirectional bladelet negatives. The way in which the blank was fractured is not identifiable: the back is completely retouched by short removals, semi-abrupt to abrupt, forming very pointed ends. The opposed edge, left untouched, is slightly convex. This segment is unique in the North square. Only one additional segment was collected in stratigraphy, in extension 1 of section NExt-4: this artefact with broken extremities is very similar in terms of dimension and techniques (3.2 cm long, 1.8 cm wide and 0.7 cm thick) (Table 7, Fig 19, n°2). Several removals could correspond to use-wear or impact scars on the edge.

**Table 7:** Dimensions of the two segments found in stratigraphy and the five segments collected on surface.

	Length (cm)	Width (cm)	Thickness (cm)
Segment 1 (North square)	3.5	2.2	0.7
Segment 2 (Next-4)	3.2	1.8	0.7
Segment 3 (surface)	3.9	1.8	0.5
Segment 4 (surface)	3.4	1.6	0.5
Segment 5 (surface)	4.0	1.6	0.9
Segment 6 (surface)	3.7	1.4	0.7
Segment 7 (surface)	5.5	2.4	0.8

It is worth noting that five segments and points were found on the surface: four of these however do not show a continuous retouched edge and are made on jaspoidal grauwacke (blue-green or brown) (Fig 19, n° 3 to 6). Their length and thickness are similar to the segments found in stratigraphy (3.4 to 3.9 cm long and 0.5 to 0.9 cm thick) but they can be narrower (1.4 to 1.8 cm wide), or more arched. The fifth is longer and wider (5.5 cm long and 2.4 cm wide), not much thicker (0.8 cm), highly weathered, but generally shares the same technical criteria as the segments found in stratigraphy (Fig 19, n° 7). Other weathered pieces have been identified in Concentration 1, but it is not possible to conclude on their relationship to the stratified artefacts. The differences in production method, dimensions, raw material and/or surface condition prevent any connection between the two groups.

Concerning the two segments found in stratigraphy and taking into account the retouching of the back, the blanks used probably measure a minimum of 2 cm wide, which does not correspond to the widest bladelet shown in Fig 16 (1.9 cm maximum). We can therefore consider at least two hypotheses. The first hypothesis is that the segments abandoned on the site are the result of *chaînes opératoires* that are totally distinct from the production carried out at Fatandi V. This assumption is supported by the difference in raw material between segments and debitage blanks. However, the segments found on the surface and made of grauwacke, but with more varied dimensions, can complicate this model. The second hypothesis considers a production on site of wider bladelets or elongated flakes in the first phases of core debitage and then a retouching of these blanks in segments. These tools would then be taken, used and abandoned elsewhere.

Regarding the use of these segments, the location of the breaks observed on the NExt-4 segment could suggest a projectile use (Fig 19, n°2). However, without a use-wear study and/or a ballistic analysis, or even experimentation with the raw materials concerned, the interpretation of the use is a speculative matter. A preliminary observation showed the probable presence of traces that could be studied in use-wear analysis.

The other retouched artefacts were made on a flake (N = 1), a bladelet (N = 1) or a natural blank (N = 4). Two pieces remain indeterminate. The retouch is marginal and short on the flake and on the bladelet (Fig 19, n°9). The natural blanks are small in size. The retouch on these pieces is abrupt and is located generally on one convergent edge (Fig 19, n°8). At least one of the artefacts presents a blunted edge on the retouched section.

#### Miscellaneous (Hematite and hammerstones)

**Fig 20:**
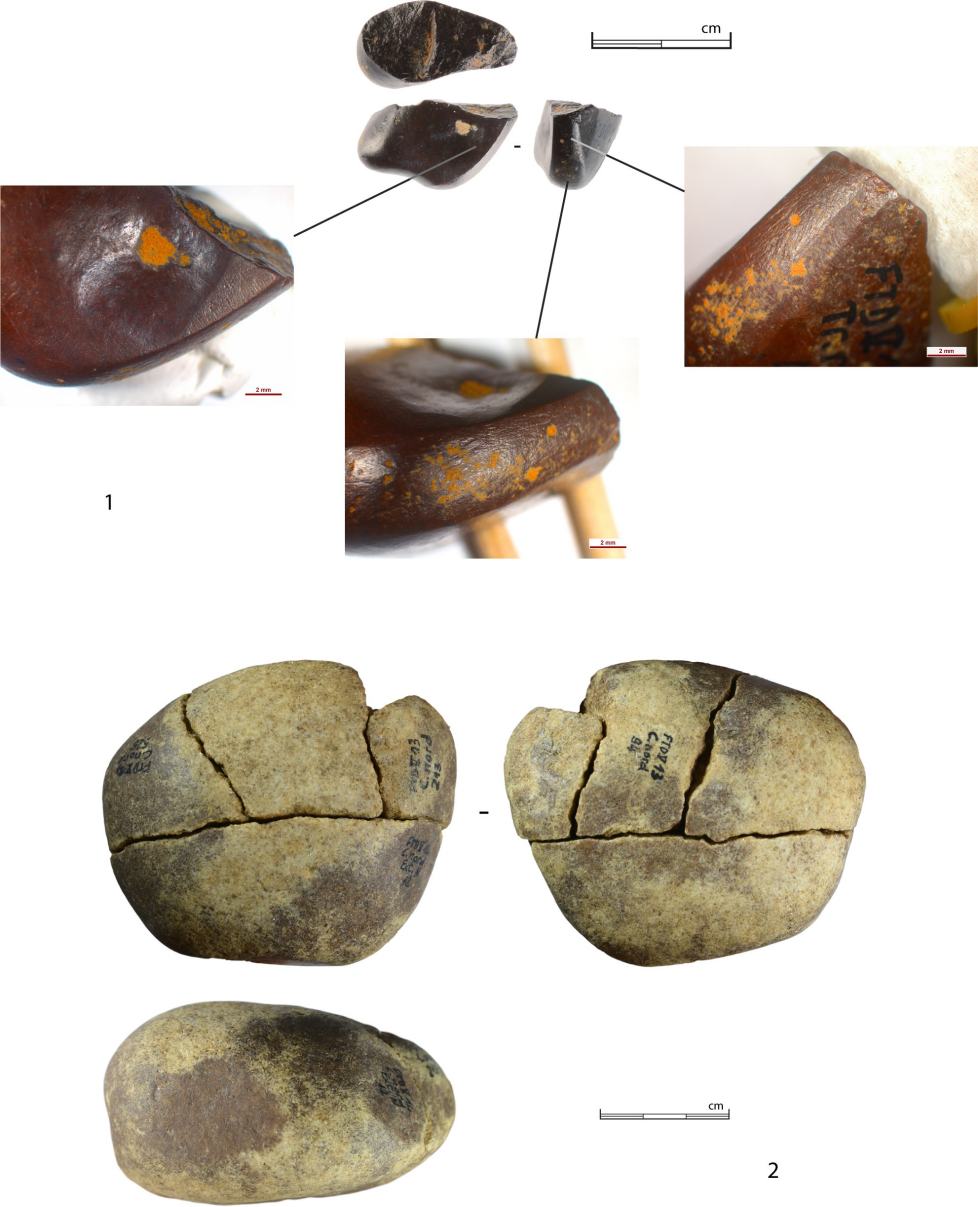
Miscellaneous artefacts. 1: Small hematite pebble, fractured and striated. 2: Fractured sandstone pebble with hammered surfaces (photographs B. Chevrier).

It is also worth noting two non-knapped elements. The first is a sandstone pebble, broken in at least four fragments, discovered in the North square (at least one piece was missing at the time of excavation). Its function as a hammerstone is demonstrated by the numerous hammered surfaces (Fig 20, n°2). In the North trench, a small hematite pebble was recovered. It measures about 23 by 12 mm. It is broken and the fractured surface shows traces of bipolar debitage (lancets and opposed impact points). Several flat faces of this pebble are striated (Fig 20, n°1). These striations are mostly parallel, with a small amount being orthogonal. They could predate the bipolar fragmentation, but only a microscopic analysis could prove this. It is currently impossible to determine its use and no evidence of hematite has been observed on the lithic artefacts.

## Discussion

### The site of Fatandi in its palaeoenvironmental context

The glacis were built up during the Upper Pleistocene [2,10–11,22,48] and form the geomorphological context for later evolution. The last dry and cool period of the Upper Pleistocene attested in tropical Africa corresponds to the Younger Dryas [68,69]. In Sudano-Saharan western Africa, this period is characterized by an increase in dust circulation flows, well documented south of the Sahara. These flows reduce again around 9,700–9,600 BCE [11.7–11.6 ka BP] [70,71], during which this climatic event ceases on a global scale [72]. Subsequently, palaeoenvironmental data recovered from the banks of the Falémé at Fatandi show two successive phases at the end of Pleistocene and beginning of Holocene. During the first period (13,400–8,200 BCE [15.4–10.2 ka at 68%, 16.9–9.4 ka at 95%]), silty to sandy sediments appear, representing a mixture of flood silts from the Falémé and wind-blown sediments moved by surface runoff. These sediments indicate the continuation of sedimentary processes observed during the last millennia of the Upper Pleistocene, although the pedogenesis affecting the sediments is an original feature indicating cooler conditions than those of today and a climate of contrasting seasons. The seasonality of the climate may be related to the northward shift of the monsoon after the Younger Dryas, reaching the 14^th^ parallel around 9,500 BCE [11.5 ka] [73]. Nevertheless, local climatic conditions appear to be even cooler than today and the relatively open vegetation is characterized by a low grassy layer, suggesting drier conditions. Then, the second recorded phase shows that the woody environment becomes denser and that the gallery forest of Bambusoideae and Arecaceae develops in parallel with an iron-rich pedogenesis process. This indicates the first long-term consequences of the beginning of global warming and rainfall that characterize the West African hydrological optimum [68,74,75]. It also corresponds to the rapid rise in the level of Lake Chad [76]. It is in this transitional environment between the open spaces of the glacis and the plateau, and the increasingly wooded alluvial corridor, that the populations of Fatandi settled. These humid zones, which can be temporarily submerged during the rainy season, must have been particularly attractive during the dry season because of their rich resources (raw materials, water, trees, and bushes).

A comparison with data obtained a little further north, in the current Sahelian environment at Ounjougou (14.2°N), provides a rich source of information on the geography of climatic improvement in the Early Holocene [37]. Indeed, abundant hydrological activity produces energetic and seasonal flooding events. It was not before 9,400–8,700 BCE [11.4–10.7 ka] that these events became more regular, although seasonality remains well recorded until about 8,200 BCE [10.2 ka], before the reemergence of energetic flooding events around 7,000 BCE [9 ka] [77]. In parallel, the re-invasion of vegetation first appeared through the transition from an open landscape with denser riparian vegetation (9,400–8,700 BCE [11.4–10.7 ka]) to a later period (8,700–8,200 BCE [10.7–10.2 ka]) with the development of wooded and shrubby vegetation. This vegetation indicates a relative closing of the landscape and the consolidation of a savanna landscape characterized by xerophytic grasses [37,78]. At Fatandi, the vegetation is nevertheless more closed than at Ounjougou during this second phase, as evidenced by the presence of Bambusoideae, which today only grow in the Sudano-Guinean zone. Thus, from the beginning of the Holocene, the northward progression of the monsoon front, biomes and bioclimatic zoning was established for the whole of Sudano-Sahelian Africa. According to Collins *et al*. 2013 [70], the position of the border between the Saharan and Sahelian environments is located around 19°N at about 8,000 BCE [10 ka]. However, the environmental dynamics observed at Fatandi show that within the Sudanese environments, the development of wooded vegetation was a complex process. Indeed, a gradient of landscape closure and riparian forest diversity can be observed at the beginning of the Holocene within the Sudanian zone of the period. In contrast to the environment exploited by populations practicing intensive foraging and ceramic production at the beginning of the Holocene at Ounjougou [4], the human groups at Fatandi exploited an environment with denser and more diverse riparian vegetation and alluvial plains with regular flooding.

### Taphonomic, chronological and archaeological synthesis

Taphonomic and archaeological analysis tend to indicate the existence of a single archaeological layer, well preserved in stratigraphy in the northern half of the site. It consists of several dense and more or less clearly delimited lithic concentrations, which most probably represent one or multiple short-term occupations. OSL analyses have allowed us to locate this layer at the end of the Pleistocene or at the beginning of the Holocene. The artefacts were collected in the lower part of U2 or at the interface of U1 and U2, respectively dated from 13,400 to 8,200 BCE [15.4–10.2 ka at 68%, 16.9–9.4 ka at 95%] and 10,900 to 7,500 BCE [12.9–9.5 ka at 68%, 14.2–8.7 ka at 95%]. The only OSL sample collected directly from the archaeological layer in the northern part of the site confirms this chronological framework and occupation at the end of Pleistocene or the very beginning of the Holocene: 11,300 to 9,200 BCE [13.3–11.2 ka at 68%, 14.3–10.3 ka at 95%].

The sample of lithic material from the very well-preserved concentration in the North square, supplemented by several artefacts from adjacent sectors, together show the excellent state of preservation of the artefacts. These artefacts were made almost exclusively out of a blueish-green grauwacke of high quality, despite the numerous fissures. The assemblage is mainly oriented towards the production of bladelets. Several methods have been identified but all of them are simple in their execution, with very short series of production. The produced blanks seem to be mainly thin, somewhat wide, rectilinear bladelets. These productions are associated with flake production, also of simple conceptual process. Several bladelet cores are occasionally recycled into flake cores, according to this principle. The tools are few in number, consisting of several roughly retouched blanks, but also of two segments (in stratigraphy) executed on a different, more chalcedoneous raw material. These segments seem to have been produced on bladelet or elongated blanks. These blanks are technically similar to those made from the bladelet productions identified in the assemblage, but their dimensions seem to be slightly larger. This observation and the difference in raw material raise the question of abandonment and export of segments made on site.

The function of the site currently remains difficult to establish. The absence of fauna and organic remains is typical of West African contexts. Due to the sedimentary conditions, which are not favorable to the preservation of this type of remains, an important part of the information is lost. The presence of multiple lithic concentrations, composed of several hundreds or even thousands of artefacts, attests to nearly-complete *chaînes opératoires*. However, the blanks that could be retouched such as the segments we collected seem to be missing, and tools made of non-local raw material have been abandoned. While this appears to be evidence of knapping operations on the site of Fatandi V, it would be premature to conclude that a knapping workshop was present. Indeed, several factors complicate this interpretation. The concentrations of numerous artefacts may suggest intensive knapping activity. However, the raw material used is extremely fissured and many artefacts represent unused blocks or natural fragments and splitted debris. Moreover, since the industries were very simple and short series production, the waste thus seems overly abundant compared to the final result (several dozen blanks). It should also be noted that the stratified concentration in the North square, with its 729 artefacts longer than 2 cm, closely concentrated in an area less than 1m^2^, could represent a waste disposal zone rather than a true knapping area. The management of this space could therefore be essential to understanding the organization and functioning of the site. The presence of a small striated hematite pebble also raises questions: was it used for knapping purposes, or for another activity carried out at the site? The scarcity of the retouched tools raises the question of other activities that may have been performed at the site. Finally, as the raw material was not available in the immediate vicinity, the blocks of grauwacke had to be selected and transported to Fatandi from elsewhere, which would justify the site being something other than a knapping site. Only an extensive excavation coupled with a spatial analysis of the other well-preserved areas of occupation, as well as a micro-stratigraphic analysis of the knapping piles, could provide relevant information.

### Comparison with MIS 2 and Holocene sites

This site has produced first data relating to the occupation of the Falémé and, more generally for the basin of the river Senegal, during the end of Pleistocene and Early Holocene. It clearly features a technical tradition anchored in bladelet industries with geometric segments. As regards close sites for the same period, the site of Toumboura I, located about 12 km north of Fatandi V (Fig 1), yields more than 1,200 lithics in a very well stratified layer. OSL sampling dated the layer to 14,000–12,000 BCE [15 ± 1 ka], somewhat earlier than Fatandi V [2,79]. The raw material used in Toumboura I is mainly the same gauwracke than the one at Fatandi V, but the blocks are smaller with alluvial cortex: the place of procurement might differ. *Chaînes opératoires* are also different with unipolar, bipolar and orthogonal series of debitage to obtain small flakes and bladelets. Abrupt retouching is made to produce numerous segments and points, never longer than 2 to 3 cm. These elements are associated with larger flakes made on siliceous rock and sandstone by hard percussion, and no ceramics or organic remains were found. Since certain technical differences are evident, in particular with regards to the type of blanks selected, as well as the types of armatures and their dimensions, we must remain prudent regarding a potential cultural link between Toumboura I and Fatandi V. But the use of segments and the absence of ceramics connect these two sites in a wide range.

The site of Ravin des Guêpiers, in the Falémé valley too, is chronologically earlier, attributed to mid-MIS 2, and technologically very different with very simple debitage methods [2,79]. Recently, in Sansandé sediments, 10 km north of Fatandi (Fig 1), a new archaeological layer has yielded a LSA lithic assemblage with flake and blade/bladelet debitage, associated with segments, endscrapers and denticulates [80]. It is deposited under a layer with ceramics. Preliminary chronological analysis suggests a more recent date than in Fatandi V: it could be a Middle Holocene occupation, at the end of 6^th^ millennium BCE.

In the Senegal valley, north of the country, a new site has also recently been identified, Ndiayène Pendao [81]. Its archaeological composition is clearly of MSA type (Levallois methods, core axes, basally thinned flakes etc.) but the OSL dating attributes the assemblage to the Pleistocene/Holocene transition. If the age is confirmed, this would indicate a very late extension of the MSA cultural tradition. However, the questions that can be raised regarding dating (only two OSL samples in relation to the stratified artefacts, a stratigraphic inversion between two dates with about 11ka difference, the choice for the most recent date) limit us for a comparison between Fatandi V and Ndiayène Pendao. More stratigraphic and geochronological data are needed for this site.

More broadly, considering only the material composition of the assemblages, bladelet production and segments are known from MIS 3 at sites such as Shum Laka in Cameroon [6] to the 2^nd^ millennium BCE [2^nd^ mill. cal BC] at Fanfannyégèné 1 [82]. It is not possible to exclude with certainty the use of ceramics and grinding material among the populations of Fatandi, as the absence of such objects could simply be due to the function of the site. We are nonetheless able to exclude the use of bifacial shaping techniques for the production of armatures. This reality in terms of the composition of assemblages leads us to propose the existence of at least two clearly distinct groups in western Africa from the very end of Pleistocene or beginning of the Holocene.

The first group is composed of assemblages with exclusively geometric armatures. Beginning in the 13^th^ millennium BCE [13^th^ mill. cal BC], this technical choice is initially made in the absence of ceramics or grinding material, as we also see at Iwo Eleru in Nigeria, Bingerville in the Ivory Coast, or at Damatoumou—Ounjougou in Mali [5,83,84]. Fatandi V and Toumboura I could fit this description. Without ruling out the idea of a functional option within a territorial set of sites of the same group, or simple technical convergences, we put forward the hypothesis that these populations may belong to the cultural lineage of Shum Laka, identified in south-western Cameroon, with a sequence that extends from the end of MIS 3, from 30,000 to 8,000 BCE [32,000 ka to 10,000 ka] [6]. This first group is attributed to the LSA and named by K.C. MacDonald the *West African Microlithic Technocomplex* [7]. While these different populations share a common conception of geometric armatures, this phenomenon would deserve to be studied more closely in order to identify potential differences in the management of the blanks (types, dimensions etc.). In the current state of research, the site of Ravin du Hibou—Ounjougou in Mali is the earliest known site where, during the 8^th^ millennium BCE [8^th^ mill. cal BC], ceramics and grinding material (grindstone and hand grinders) are directly associated with a microlithic industry comprising segments [85]. This triple association seems to appear a little later in southern Mali, such as at Kourounkorokalé around 4,000 BCE [4,000 cal BC] [7]. This tradition persists in some places until the 2^nd^ millennium BCE [2^nd^ mill. cal BC] on the Senegalese coast or at Fanfannyégèné I in Mali [82,86].

The second group is characterized by the use of bifacial armatures, accompanied in its initial phase by ceramics (or the stoneware of Temet) and grinding material [4,87–89], and is recognized in different places in western Africa. These first signs currently date back to the 10^th^ millennium BCE [10^th^ mill. cal BC] in Mali at Ravin de la Mouche—Ounjougou, or to the 9^th^ millennium BCE [9^th^ mill. cal BC] in Niger, as at the Saharan sites of Tagalagal and Temet. If the differences between the two groups are of a cultural nature and if the use of geometrics really exclude the production of bifacial armatures, these populations may evolve in alternation, as at the site of Le Promontoire—Ounjougou between the 6^th^ and 4^th^ millennium BCE [6^th^ and 4^th^ mill. cal BC] in Mali [8], within the different Neolithic groups at Tilemsi in the north of Mali from the beginning of the 3^rd^ millennium BCE [3^rd^ mill. cal BC] [90,91] or at Windé Koroji from the beginning of the 2^nd^ millennium BCE [2^nd^ mill. cal BC], or north of Ounjougou, still in Mali [92]. On the other hand, further south, in Ghana, a complete cultural ensemble, called Kintampo culture, as seen at Ntereso, is characterized by this same type of bifacial armatures accompanied by ceramics reminiscent of certain features of southern Sahara ceramics, such as those of Kobadi [93–95].

The environmental data collected at Ounjougou and Fatandi could in theory suggest environmental factors as an element for distinguishing cultural practices and material industries. Nevertheless, throughout a large part of the Holocene, there is a real juxtaposition of Saharan and sub-Saharan populations with one or another of these kinds of armatures. They evolved either in alternation within the same region (as at Ounjougou), or in parallel (as in Ghana, where the Kintampo culture was established within the context of microlithic cultural groups). This allows us to consider here the hypothesis that two completely techno-culturally distinct populations coexisted at the very end of Pleistocene and during the Holocene in western Africa. This suggests eliminating the role of the environment in the mobility of these populations from the beginning of the Holocene. After the first phases, which remain to be studied, these populations were no longer dependent on a specific ecological niche. Of course, this opposition is macro-cultural and macro-geographic [96]. This proposal does not preclude the existence of finer techno-cultural differences within each group. This model still needs to be tested and strengthened by more detailed technological studies on all the sites concerned, especially on sites investigated several decades ago, in connection with accurate geochronological and palaeoenvironmental data. A comparison with anthropological and genetic knowledge would also make it possible to compare the different available data.

## Conclusion

The Fatandi V site constitutes the first stratified site around the Pleistocene/Holocene boundary in Senegal with both precise geochronological and palaeoenvironmental data. It perfectly complements the data already obtained in Mali and in the rest of western Africa, and thus constitutes a reference point for this period at the end of Pleistocene and beginning of the Holocene. In any case, the assemblage of Fatandi V, with its bladelet debitages and segments and in the absence of ceramics and grinding material, is related to a group using exclusively geometric armatures, which strongly differs from another group characterized by the production of bifacial armatures, accompanied in its initial phase by ceramics (or stoneware) and grinding material. A cultural distinction is hypothesized. The available geographical and palaeovironmental data prevent any geographical determinism in the spatial distribution of these two groups. Moreover, the pattern of the excavated sites attributed to these groups in West Africa does not suggest any specific adaptation to environmental change. Thus, the distinction between two cultural groups could indicate a complex spatio-temporal pattern of cultural origin that remains to be studied and understood. We lack data to measure the significance of this cultural model, but this is probably a crucial question to better understand the settlement pattern of West Africa from the Late Stone Age to the Early Neolithic and the development of farming and herding practices. For the time being, the weakness of surveys and excavations focused on this type of well stratified and preserved archeological sites prevents a better understanding of this complex situation. Therefore, further fieldwork and archaeological research need to be supported to explain the transition from the Late Stone Age and foraging groups to the productive societies in West Africa.

## Supporting information

S1 Fig Comparison of the specific activity from the “head” (^238^U determined according to the readings for ^234^Th, ^234m^Pa, and ^235^U), in the “middle” (^226^Ra determined from ^214^Pb and ^214^Bi) and at the “bottom” of the chain (^210^Pb) of uranium decay. The error is given to 1σ. The samples with an asterix are those that were processed by Lebrun *et al*. (2016) [23] and mentioned here as a reminder.
(TIF)

S2 Fig Example of the preheat plateau obtained for sample F3. (TIF)

S3 Fig Representation of the value of the De for each sample according to the statistical model employed. The equivalent doses are normalised with regards to the average of the estimations of different single-grain models. The error ranges or credibility interval are not represented here for sake of clarity. Blue circles: multi-grain CAM; green diamonds: single-grain CAM; green circles: baSAR-LogNormal_M; orange circles: baSAR-Normal; red diamonds: arithmetic mean.
(TIF)

S1 File R Codes applied for the Bayesian analyses of the equivalent dose measurements and age calculations. (PDF)

S2 File (DOCX)
