# Supplementary figures and images for: New data on settlement and environment at the Pleistocene/Holocene boundary in Sudano-Sahelian West Africa: Interdisciplinary investigation at Fatandi V, Eastern Senegal
Source: PLoS One. 2020 Dec 9;15(12):e0243129. doi: 10.1371/journal.pone.0243129 (PMC7725507; doi:10.1371/journal.pone.0243129)

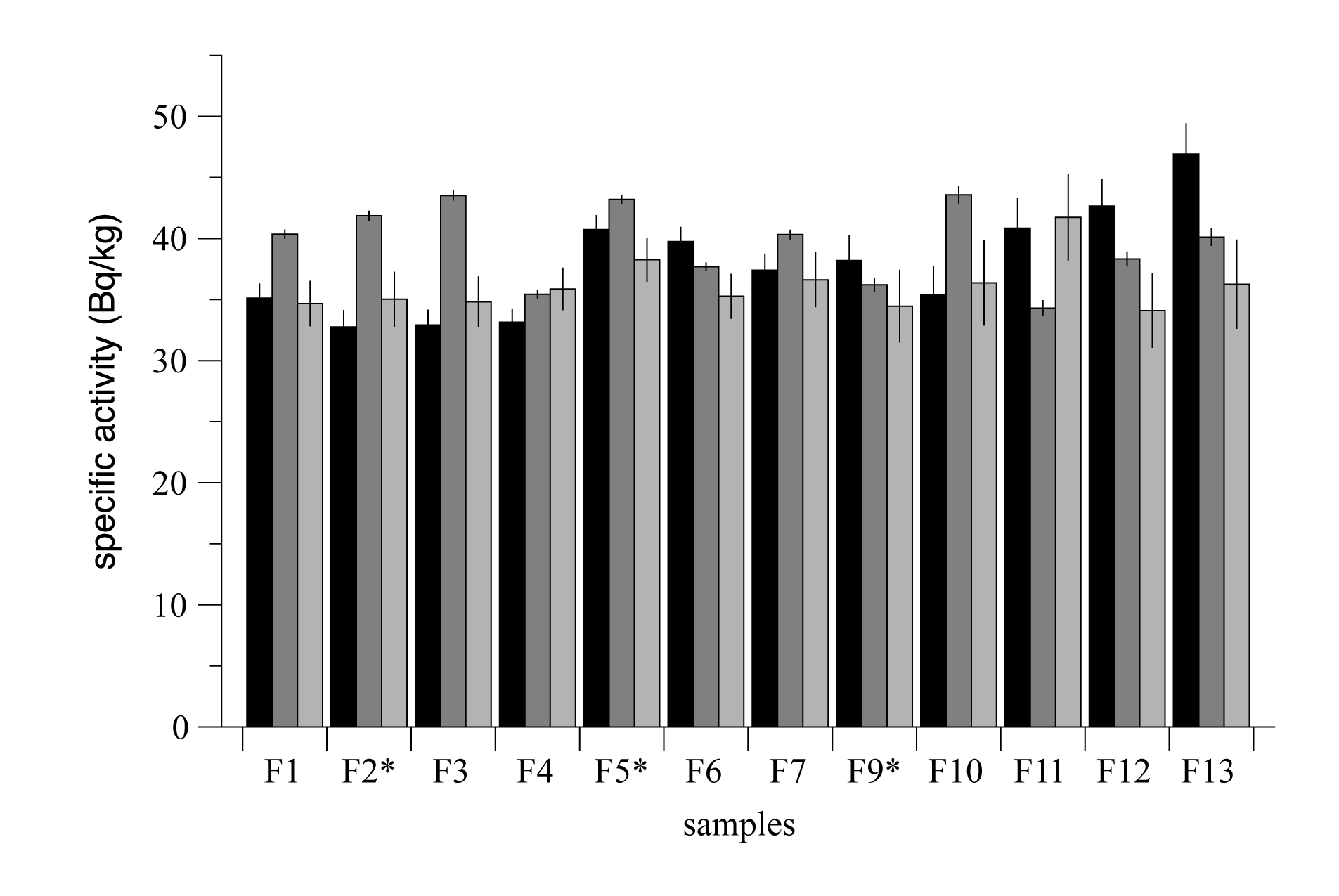

Supplement: S1 Fig — The error is given to 1σ. The samples with an asterix are those that were processed by Lebrun et al. (2016) [23] and mentioned here as a reminder. (TIF) [file pone.0243129.s001.tif]

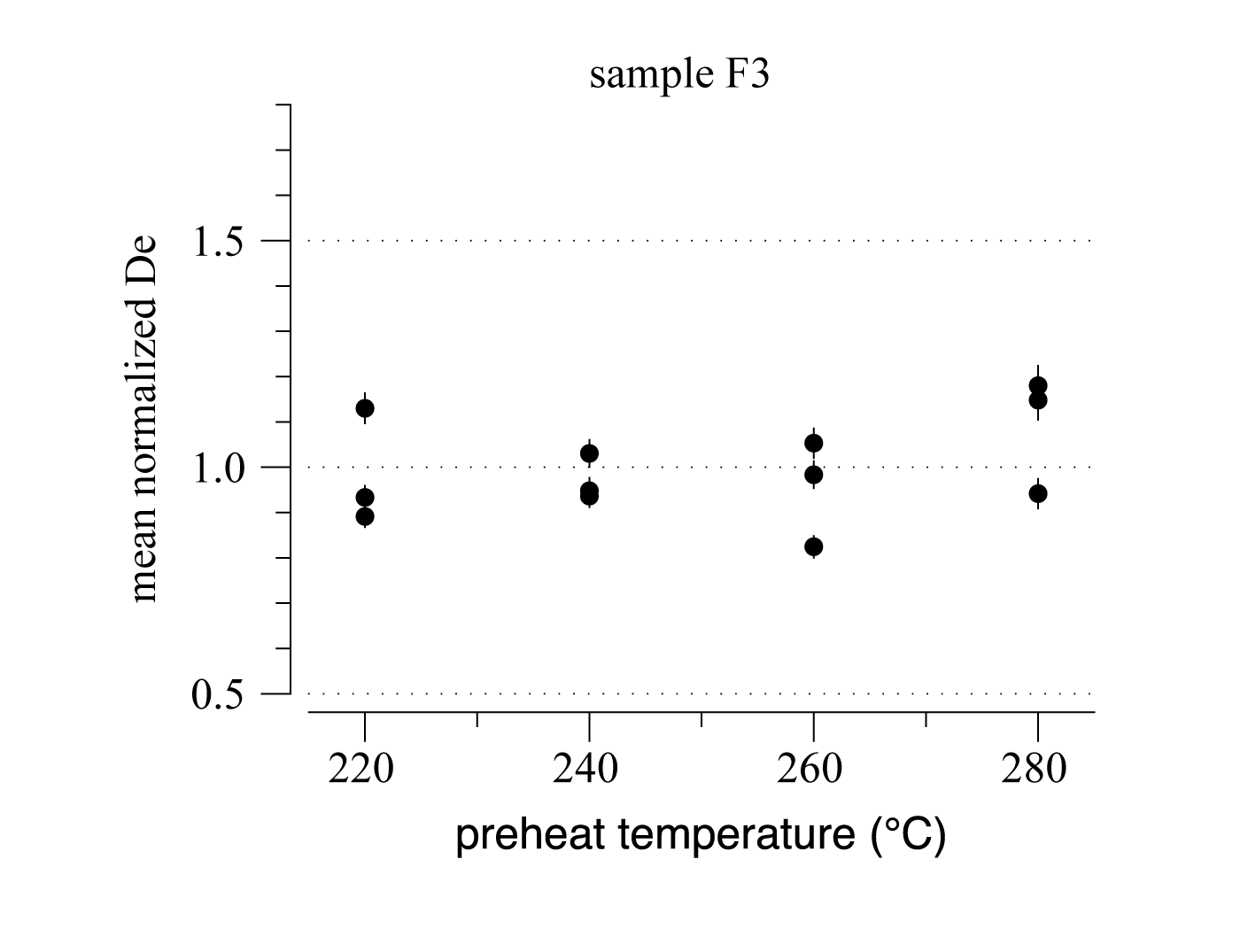

Supplement: S2 Fig — (TIF) [file pone.0243129.s002.tif]

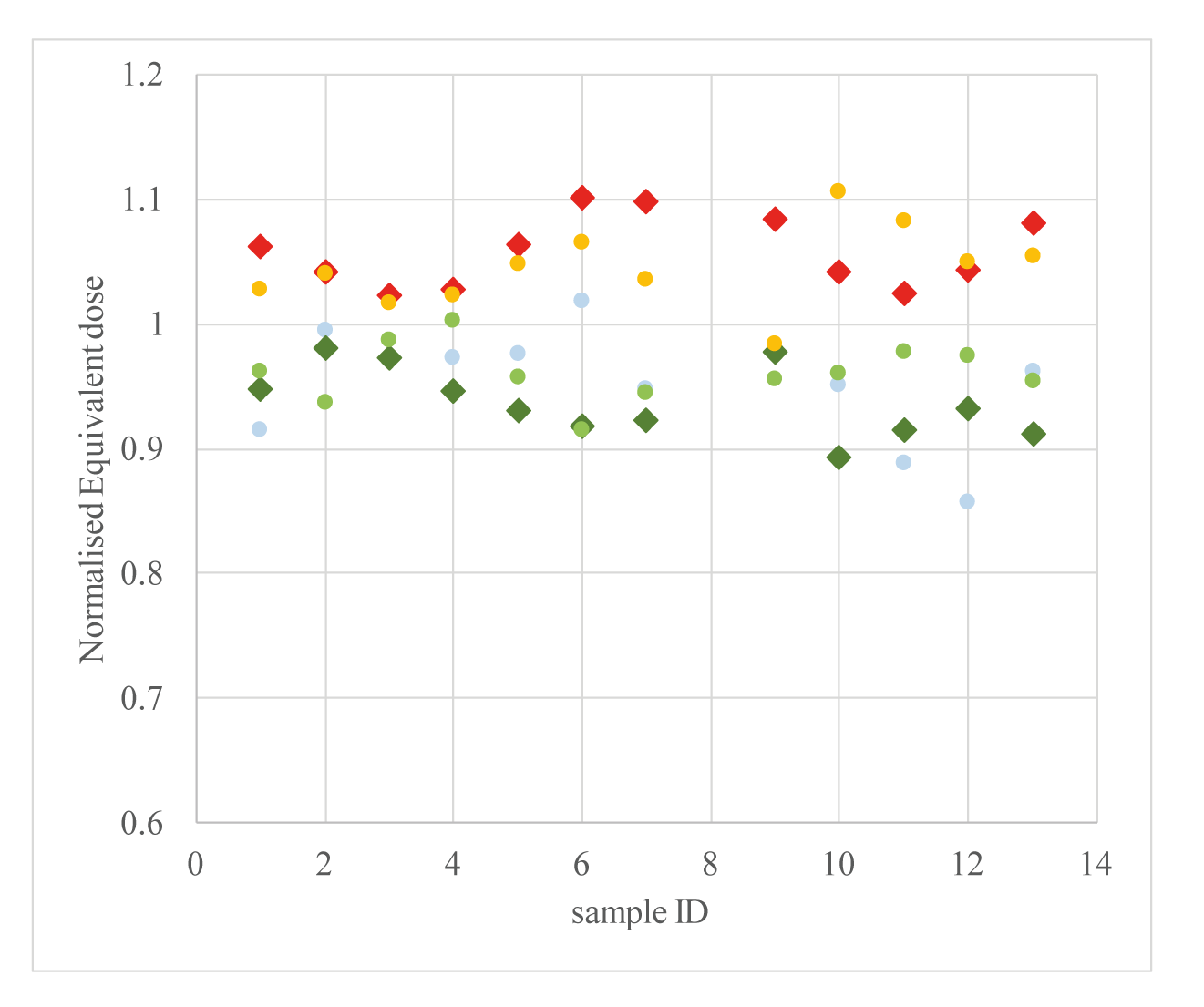

Supplement: S3 Fig — The equivalent doses are normalised with regards to the average of the estimations of different single-grain models. The error ranges or credibility interval are not represented here for sake of clarity. Blue circles: multi-grain CAM; green diamonds: single-grain CAM; green circles: baSAR-LogNormal_M; orange circles: baSAR-Normal; red diamonds: arithmetic mean. (TIF) [file pone.0243129.s003.tif]
