# Supplementary material for: New data on settlement and environment at the Pleistocene/Holocene boundary in Sudano-Sahelian West Africa: Interdisciplinary investigation at Fatandi V, Eastern Senegal
Source: PLoS One. 2020 Dec 9;15(12):e0243129. doi: 10.1371/journal.pone.0243129 (PMC7725507; doi:10.1371/journal.pone.0243129)
Supplement: S1 File — (PDF) [file pone.0243129.s004.pdf]

S1 File. R Codes applied for the Bayesian analyses of the equivalent dose measurements and age calculations. They are based on: Combès and Philippe, 2018; Christophe *et al.*, 2018; Philippe *et al.*, 2019; Philippe and Vibet, 2017, 2019 (references at the end of the file).

Dose recovery tests: Bayesian model assuming a Gaussian distribution or a lognormal distribution with calculation of the median.

Note: a folder must be prepared with:

- The bin file prepared so that all needed luminescence records (Lx and Tx) are set to "true" and the others to "false".
- A csv file where the selected grains are indicated. Compulsary name: "DicsPos", two columns, headers :"position" and "grain"; separator: comma.
- A csv file where named "DoseEnv", two columns, headers: "obs", "var"; separator: comma. For the dose recovery test, "obs" must be equal to the dose to recover in seconds divided by 1000 and var <<obs.
- A csv file named: "DoseSource", two columns, headers: "obs", "var"; separator: comma. For the dose recovery test, obs=0.1 and var=0.00001
- A csv file where the basic rules for analysis are indicated (integration channels, use of the recycling point, reproducibililty), e.g.:

```
[Param]
beginSignal= 6
endSignal= 15
beginBackground= 50
endBackground= 55
beginTest= 6
endTest= 15
beginTestBackground= 50
endTestBackground= 55
inflatePercent= 0.025
nbOfLastCycleToRemove= 1
```

Note : For a dose recovery ratio of 1, the output here will be 10.

Here we give the example of sample F4.

```
### ============= preparation


rm(list=ls())   # suppress previous objects

library(BayLum) # package loading

library(rjags)
```

```
### ============= variable informations

Path <- c("DRTfile/") #shows where the bin file and associated csv files are located
SavePath <- c("resultsDRT/DRT-F4/F4-3000kit")  #shows where the results will be stored
Names <- "F4-DRT" # name of the file where the bin files are stored

### ============= generate Datafiles

DATA <- Generate_DataFile(Path,
                          FolderNames = "F4-DRT",
                          Nb_sample = 1,
                          Nb_binfile = 1,
                          BinPerSample =  1)

save(DATA, file = "DATA.RData") # record of the Datafile

### ============= calculation of the dose recovery ratios
A <-
  Age_Computation(
    DATA,
    SampleName = "F4",
    PriorAge = c(1,25),
    BinPerSample = 1,
    SavePdf = TRUE,
    OutputFileName = c("resultatBayesien"),
    OutputFilePath = SavePath,
    SaveEstimates = TRUE,
    OutputTableName = c("resultatBayesien"),
    OutputTablePath = SavePath,
    LIN_fit = FALSE,
```

```
    Origin_fit = TRUE,
    distribution = c("gaussian"),
    Iter = 3000000,
    t = 5,
    n.chains = 3
  )
```

here the code holds for a Gaussian distribution; for a lognormal distribution and median,
#“gaussian” must be replaced by “lognormal_M”

Equivalent dose: Bayesian model assuming a Gaussian distribution or lognormal distribution and the age equal to the median; no stratigraphic constraints

---

Note: a folder must be prepared with:

- The bin file prepared so that all needed records (Lx and Tx) are set to “true” and the others to “false”
- A csv file where the selected grains are indicated. Name: “DicsPos”, two columns, headers: “position” and “grain”; separator: comma.
- A csv file where the environmental dose rate and its variance are indicated. Name: “DoseEnv”, two columns, headers: “obs”, “var”; separator: comma.
- A csv file where the artificial dose rate and its variance are indicated. Name: “DoseSource”, two columns, headers: “obs”, “var”; separator: comma
- A csv file where the basic rules for analysis are indicated (integration channels, use of the recycling point, reproducibililty), e.g.:

```
[Param]
beginSignal= 6
endSignal= 15
beginBackground= 50
endBackground= 55
beginTest= 6
endTest= 15
beginTestBackground= 50
endTestBackground= 55
inflatePercent= 0.017
nbOfLastCycleToRemove= 1
```

```
### ============ preparation

rm(list=ls())   # suppress previous objects
library(BayLum) # package loading
```

```
library(rjags)

### ============= variable information

Path <- c("DefileFatandi/") #shows where the bin file and associated csv files are located
SavePath <- c("resultsDe/De-F4/F4-3000kit")  #shows where the results will be stored
Names <- "F4-De" # name of the file where the bin files are stored



### ============= generate Datafiles

DATA <- Generate_DataFile(Path,
                          FolderNames = "F4-De",
                          Nb_sample = 1,
                          Nb_binfile = 1,
                          BinPerSample =  1)

save(DATA, file = "DATA.RData") # record of the Datafile

### ============= calculation of the ages
A <-
  Age_Computation(
    DATA,
    SampleName = "F4",
    PriorAge = c(1,25),
    BinPerSample = 1,
    SavePdf = TRUE,
    OutputFileName = c("resultatBayesien"),
    OutputFilePath = SavePath,
    SaveEstimates = TRUE,
    OutputTableName = c("resultatBayesien"),
```

```
    OutputTablePath = SavePath,
    LIN_fit = FALSE,
    Origin_fit = TRUE,
    distribution = c("gaussian"),
    Iter = 3000000,
    t = 5,
    n.chains = 3
  )
# here the code holds for a Gaussian distribution; for a lognormal distribution and median,
#"gaussian" must be replaced by "lognormal_M"
```

Equivalent dose: Bayesian model assuming a log normal distribution and calculation of the median or a Gaussian distribution **with** stratigraphic constraints

---

Note: we use the same folders with the bin and csv files as for the determination of the ages without stratigraphic constraints.

In addition, a matrix showing the covariances between the systematic errors of the different samples must be prepared (see Heydari et al., in press (supplementary materials) for detailed explanation on the construction of the matrix and Combès and Philippe, 2017). The matrix is called "ThetaFatandi.csv" and is stored in the working directory.

```
### ============= preparation
rm(list=ls())   # supress previous objects
library(BayLum) # package loading
library(ArchaeoPhases)
library(rjags)
library(DT)


### ============= variable information


Path <- c("DeFatandi/") # shows where the bin files and associated csv files are located
SavePath <- c("resultsDewithStratigraphicConstraints/")  # shows where the results will be stored
Names <- c(
```

```
  "De-F7-SG","De-F6-SG","De-F5-SG","De-F13-SG","De-F12-SG","De-F10-SG","De-F9-SG","De-F4-
SG","De-F3-SG","De-F2-SG","De-F1-SG","De-F11-SG")

# name of the files where the bin files are stored


### ============ generate Datafiles


DATA_tous <- Generate_DataFile(Path,

                         FolderNames = c("De-F7-SG","De-F6-SG","De-F5-SG","De-F13-SG","De-F12-SG","De-
F10-SG","De-F9-SG","De-F4-SG","De-F3-SG","De-F2-SG","De-F1-SG","De-F11-SG"),

                         Nb_sample = 12,

                         Nb_binfile = 12,

                         BinPerSample =  c(1,1,1,1,1,1,1,1,1,1,1,1),

                         verbose = TRUE)


# creation of a single file

save(DATA_tous, file = "DATA.RData")


### ============ creation of the matrix indicating the stratigraphic constraints


SC = matrix(data=0,ncol=12,nrow=13) # basic definition of the matrix

SC[1,]=c(1,1,1,1,1,1,1,1,1,1,1,1)   # lower boundary, applies to each sample

SC[2,]=c(0,1,1,1,1,1,1,1,1,1,1,1)   # F7

SC[3,]=c(0,0,1,1,1,1,1,1,1,1,1,1)   # F6

SC[4,]=c(0,0,0,1,1,1,1,1,1,1,1,1)   # F5

SC[5,]=c(0,0,0,0,1,1,1,1,1,1,1,1)   # F13

SC[7,]=c(0,0,0,0,0,1,1,1,1,1,1,1)   # F12

SC[6,]=c(0,0,0,0,0,0,1,0,0,0,0,0)   # F10

SC[8,]=c(0,0,0,0,0,0,0,0,0,0,0,0)   # F9

SC[9,]=c(0,0,0,0,0,0,0,0,1,0,0,0)    # F4

SC[10,]=c(0,0,0,0,0,0,0,0,0,0,0,0)  # F3

SC[11,]=c(0,0,0,0,0,0,0,0,0,0,1,0)  # F2

SC[12,]=c(0,0,0,0,0,0,0,0,0,0,0,0)  # F1
```

```
SC[13,]=c(0,0,0,0,0,0,0,0,0,0,0,0)  # F11

### loading of the covariance matrix

Theta = as.matrix(read.csv("ThetaFatandi.csv", header = TRUE, sep = ";"))

### ============= calculation of the ages

A <-
  AgeS_Computation(
    DATA_tous,
    SampleNames = c("nF7","mF6","lF5","kF13","jF12","iF10","bF9","fF4","cF3","eF2","dF1","aF11"),
    Nb_sample =12,
    PriorAge = rep(c(0.5, 50),12),
    BinPerSample = c(1,1,1,1,1,1,1,1,1,1,1,1),
    SavePdf = TRUE,
    OutputFileName = c("resultatBayesienSGFatandi1000kit"),
    OutputFilePath = SavePath,
    SaveEstimates = TRUE,
    OutputTableName = c("resultatBayesienSGFatandi1000kit"),
    OutputTablePath = SavePath,
    THETA = Theta,
    sepTHETA = c(";"),
    StratiConstraints = SC,
    sepSC = c(","),
    LIN_fit = FALSE,
    Origin_fit = TRUE,
    distribution = c("gaussian"),
    Iter = 1000000,
    t = 5,
```

```
  n.chains = 3
 )
# here with a Gaussian distribution; for a lognormal distribution and median calculation replace
#"gaussian" by "lognormal_M".

### ============= Visualisation
plot_Scatterplots(
 object = A$Sampling,
 sample_names = c("F7", "F6", "F5", "F13", "F12", "F10", "F9", "F4", "F3", "F2", "F1", "F11"),
 sample_selection = c(1:12)
)

### ============= ARCHEOPHASE

#save the object created with "AgeS_Computation"
save(A, file = "A.RDataSGFatandi")

#save MCMC
mcmc = A$Sampling[[1]]

# check convergence of MCMC chains
plot(A$Sampling)

# convertion of all ages in calendar dates
fatandi = as.data.frame(mcmc[,c(1:12)])
fatandi13 = (2013- fatandi*1000)

#export of MCMC
write.csv(fatandi13, file = "MCMCFatandi13.csv")

#creation of the phases corresponding to units 1, 2 and 3
```

```
Phase1 = CreateMinMaxGroup(fatandi13, c(6:12), name="Unite1")
Phase2 = CreateMinMaxGroup(fatandi13, c(4:5), name="Unite2")
Phase3 = CreateMinMaxGroup(fatandi13, c(1:3), name="Unite3")

# recording of min et max values for each phase in a csv file
write.csv(Phase1, file = "Unite1.csv")
write.csv(Phase2, file = "Unite2.csv")
write.csv(Phase3, file = "Unite3.csv")

# Statistic on min, max and duration of each phase
PhaseStatistics(Phase1$Unite1.Min, Phase1$Unite1.Max, level = 0.95)
PhaseStatistics(Phase1$Unite1.Min, Phase1$Unite1.Max, level=0.68)

PhaseStatistics(Phase2$Unite2.Min, Phase2$Unite2.Max, level = 0.95)
PhaseStatistics(Phase2$Unite2.Min, Phase2$Unite2.Max, level=0.68)

PhaseStatistics(Phase3$Unite3.Min, Phase3$Unite3.Max, level = 0.95)
PhaseStatistics(Phase3$Unite3.Min, Phase3$Unite3.Max, level=0.68)

#Export of the statistics

PhaseStatisticsObject <- PhaseStatistics(Phase1$Unite1.Min, Phase1$Unite1.Max, level = 0.95)
write.csv(PhaseStatisticsObject, file = "PhaseStatistics-95-Unite1.csv")

PhaseStatisticsObject <- PhaseStatistics(Phase2$Unite2.Min, Phase2$Unite2.Max, level = 0.95)
write.csv(PhaseStatisticsObject, file = "PhaseStatistics-95-Unite2.csv")

PhaseStatisticsObject <- PhaseStatistics(Phase3$Unite3.Min, Phase3$Unite3.Max, level = 0.95)
write.csv(PhaseStatisticsObject, file = "PhaseStatistics-95-Unite3.csv")

PhaseStatisticsObject <- PhaseStatistics(Phase1$Unite1.Min, Phase1$Unite1.Max, level = 0.68)
```

```
write.csv(PhaseStatisticsObject, file = "PhaseStatistics-68-Unite1.csv")

PhaseStatisticsObject <- PhaseStatistics(Phase2$Unite2.Min, Phase2$Unite2.Max, level = 0.68)
write.csv(PhaseStatisticsObject, file = "PhaseStatistics-68-Unite2.csv")

PhaseStatisticsObject <- PhaseStatistics(Phase3$Unite3.Min, Phase3$Unite3.Max, level = 0.68)
write.csv(PhaseStatisticsObject, file = "PhaseStatistics-68-Unite3.csv")

# Time ranges
PhaseTimeRange(Phase1$Unite1.Min, Phase1$Unite1.Max, level = 0.95)
PhaseTimeRange(Phase1$Unite1.Min, Phase1$Unite1.Max, level=0.68)

PhaseTimeRange(Phase2$Unite2.Min, Phase2$Unite2.Max, level = 0.95)
PhaseTimeRange(Phase2$Unite2.Min, Phase2$Unite2.Max, level=0.68)

PhaseTimeRange(Phase3$Unite3.Min, Phase3$Unite3.Max, level = 0.95)
PhaseTimeRange(Phase3$Unite3.Min, Phase3$Unite3.Max, level=0.68)

#export of the time ranges
PhaseTimeRangeObject <- PhaseTimeRange(Phase1$Unite1.Min, Phase1$Unite1.Max, level = 0.95)
write.csv(PhaseTimeRangeObject, file = "TimeRange95-phase1.csv")
PhaseTimeRangeObject <- PhaseTimeRange(Phase2$Unite2.Min, Phase2$Unite2.Max, level = 0.95)
write.csv(PhaseTimeRangeObject, file = "TimeRange95-phase2.csv")
PhaseTimeRangeObject <- PhaseTimeRange(Phase3$Unite3.Min, Phase3$Unite3.Max, level = 0.95)
write.csv(PhaseTimeRangeObject, file = "TimeRange95-phase3.csv")
PhaseTimeRangeObject <- PhaseTimeRange(Phase1$Unite1.Min, Phase1$Unite1.Max, level = 0.68)
write.csv(PhaseTimeRangeObject, file = "TimeRange68-phase1.csv")
PhaseTimeRangeObject <- PhaseTimeRange(Phase2$Unite2.Min, Phase2$Unite2.Max, level = 0.68)
write.csv(PhaseTimeRangeObject, file = "TimeRange68-phase2.csv")
PhaseTimeRangeObject <- PhaseTimeRange(Phase3$Unite3.Min, Phase3$Unite3.Max, level = 0.68)
write.csv(PhaseTimeRangeObject, file = "TimeRange68-phase3.csv")
```

```
# plot of the results
PhasePlot(Phase1$Unite1.Min, Phase1$Unite1.Max, level = 0.95, title = "Phase 1 - 95%")
PhasePlot(Phase2$Unite2.Min, Phase2$Unite2.Max, level = 0.95, title = "Phase 2 - 95%")
PhasePlot(Phase3$Unite3.Min, Phase3$Unite3.Max, level = 0.95, title = "Phase 3 - 95%")
PhasePlot(Phase1$Unite1.Min, Phase1$Unite1.Max, level = 0.68, title = "Phase 1 - 68%")
PhasePlot(Phase2$Unite2.Min, Phase2$Unite2.Max, level = 0.68, title = "Phase 2 - 68%")
PhasePlot(Phase3$Unite3.Min, Phase3$Unite3.Max, level = 0.68, title = "Phase 3 - 68%")
```

References

Combès B, Philippe A. Bayesian analysis of individual and systematic multiplicative errors for estimating ages with stratigraphic constraints in optically stimulated luminescence dating. Quat Geochronol. 2017;39:24-34.

Christophe C, Philippe A, Guérin G, Mercier N, Guibert P. Bayesian approach to OSL dating of poorly bleached sediment samples: Mixture Distribution Models for Dose (MD2). Radiat Meas. 2018;108:59-73.

Heydari M, Guérin G, Kreutzer S, Jamet G, Kharazian M.A, Hashemi M et al. Do Bayesian methods lead to more precise chronologies? ‘BayLum’ and a first OSL-based chronology for the palaeolithic open-air site of Mirak (Iran). Quat Geochronol. In press.

Philippe A, Guérin G, Kreutzer S. BayLum-An R package for Bayesian analysis of OSL ages: An introduction. Quat Geochronol. 2019;49:16-24.

Philippe A, Vibet M-A. Analysis of Archaeological Phases using the CRAN Package ‘ArchaeoPhases’. 2017. Available from: https://hal.archives-ouvertes.fr/hal-01347895v3

Philippe A, Vibet M-A. ArchaeoPhases: Post-Processing of the Markov Chain Simulated by ‘ChronoModel’, ‘Oxcal’ or ‘BCal’. R package version 1.4. 2020. Available from: https://CRAN.R-project.org/package=ArchaeoPhases
