# Supplementary material for: New data on settlement and environment at the Pleistocene/Holocene boundary in Sudano-Sahelian West Africa: Interdisciplinary investigation at Fatandi V, Eastern Senegal
Source: PLoS One. 2020 Dec 9;15(12):e0243129. doi: 10.1371/journal.pone.0243129 (PMC7725507; doi:10.1371/journal.pone.0243129)
Supplement: S2 File — (DOCX) [file pone.0243129.s005.docx]

Supporting Information – Figures

Figure 1A/1B:

Map design by Laboratory Archaeology and Population in Africa (APA) of the Department of Genetics and Evolution (University of Geneva) based on views from USGS Earth Resources Observatory and Science Center (<https://www.usgs.gov/centers/eros>) and CIA World Factbook (https://www.cia.gov/library/publications/the-world-factbook/).

Figure 2A/2B/2C:

Figure designed by L. Lespez based on data from fieldwork.

Figure 6:

Drawing by Benoît Chevrier (first author). No previously copyrighted.

Figure 14:

Diagram design by Laurent Lespez and Benoît Chevrier based on data from fieldwork.

Figures 15-19 and striking image:

Drawings and photographs by Benoît Chevrier. No previously copyrighted.

Figure 20:

Photographs by Benoît Chevrier. No previously copyrighted.
